# HCF-CRS: A Hybrid Content based Fuzzy Conformal Recommender System for providing recommendations with confidence

**DOI:** 10.1371/journal.pone.0204849

**Published:** 2018-10-09

**Authors:** Sundus Ayyaz, Usman Qamar, Raheel Nawaz

**Affiliations:** 1 Department of Computer Engineering, College of Electrical & Mechanical Engineering, National University of Science & Technology (NUST), Rawalpindi, Pakistan; 2 Department of Languages, Information, Communications and Journalism, Research Center for Applied Social Sciences, Centre for Advanced Computational Science, Manchester Metropolitan University, Manchester, United Kingdom; Southwest University, CHINA

## Abstract

A Recommender System (RS) is an intelligent system that assists users in finding the items of their interest (e.g. books, movies, music) by preventing them to go through huge piles of data available online. In an effort to overcome the data sparsity issue in recommender systems, this research incorporates a content based filtering technique with fuzzy inference system and a conformal prediction approach introducing a new framework called Hybrid Content based Fuzzy Conformal Recommender System (HCF-CRS). The proposed framework is implemented to be used in the domain of movies and it provides quality recommendations to users with a confidence level and an improved accuracy. In our proposed framework, first, a Content Based Filtering (CBF) technique is applied to create a user profile by considering the history of each user. CBF is useful in the situations like: lack of demographic information and the data sparsity problems. Second, a Fuzzy based technique is incorporated to find the similarities and differences between the user profile and the movies in the dataset using a set of fuzzy rules to get a predicted rating for each movie. Third, a Conformal prediction algorithm is implemented to calculate the non-conformity measure between the predicted ratings produced by fuzzy system and the actual ratings from the dataset. A p-value (confidence measure) is computed to give a level of confidence to each recommended item and a bound is set on the confidence level called a significance level ε, according to which the movies only above the specified significance level are recommended to user. By building a confidence centric hybrid conformal recommender system using the content based filtering approach with fuzzy logic and conformal prediction algorithm, the reliability and the accuracy of the system is considerably enhanced. The experiments are evaluated on MovieLens and Movie Tweetings datasets for recommending movies to the users and they are compared with other state-of-the-art recommender systems. Finally, the results confirm that the proposed algorithms perform better than the traditional ones.

## Introduction

Recommender Systems are playing an important role since 1990's [1] by solving information overload problem and assisting users by making intelligent decisions in suggesting them items of their interest [2,3]. Recommendations can be of books [4], movies [5], music [6], clothes [7] etc. Today many online stores provide recommendations to users e.g., Amazon, Netflix, Youtube etc [2].

Recommender systems are increasingly used in online environments e.g. e-commerce. It learns from the information about the customers and products and suggests suitable personalized recommendations to every customer [8]. This helps to build a significant relationship between the website and the user. Recommendation engines can also help to gain consumers’ loyalty, which is an essential business strategy in ecommerce [9]. In literature, the RSs are mainly developed using (a) Collaborative Filtering (CF) methods [10,11,12,13] and (b) Content Based Filtering (CBF) methods [13,14,15,16]. CF focuses on preferences from like-minded users and finds similar users called neighbors whose ratings strongly correlate with the current user. Eventually CF recommendation systems recommend items to user which are rated highly by its neighbors [17]. CF is divided into memory-based and model-based CF [18,19]. Based on these approaches, the similarities between items or users are calculated and the ratings by similar users are then considered to compute the predicted ratings of items for current user [20]. CF suffers from first rater or sparsity issue and cannot recommend any new item that has not been previously rated and this technique is considered useful only when there are large number of users with considerable amount of ratings in the database [21]. Content Based Filtering (CBF), on the other hand, use features (e.g. profile content) of the users or items for rating predictions [8,11,22]. It requires information like a user profile to learn user preferences and to locate and recommend items according to user preferences [19]. One of the main advantages of CBF is the main drawback found in CF, the first-rater problem. CBF, on the other hand, does not depend on having other users in the system to recommend items. Additionally, there are (c) Hybrid methods [8,11,23], that combines two or more recommendation techniques to overcome the weaknesses of any individual ones and provide recommendations with better accuracy and quality. Many algorithms have been proposed using the above-mentioned techniques but some issues are still vulnerable.

One of the current and most common issues in CF method of recommender systems is the sparsity of information related to the user cold-start problem [24]. Sparsity refers to the problem of insufficient or no ratings provided by the users to the items in the user-item database. There are two types of data sparsity situations, when there is a new item available i.e. item-based [25] or there is a new user in the system i.e. user-based [24]. The lack of user demographic data in various online systems is another issue in providing recommendations using CBF method which results in getting information either implicitly or explicitly.

Our proposed recommender system solves the sparsity issue in the database when the user-item matrix is sparse or there is new item in the database which the user has not rated yet.

We have used a Content Based Filtering (CBF) algorithm to deal with the issue mentioned above. In our proposed algorithm, CBF is used to record the user behavior implicitly and creates a user profile for each user depending upon the past browsing behavior. Movie ‘genre’ is considered one of the dominating feature in the domain of movies. Therefore, we have focused on the ‘genre’ of rated movies to create user profile and recommend items according to it. Other features such as age, occupation and gender of the user, in one case, is not always available as the user’s feel hesitant or consider time consuming for providing such information. In other case, same type of movies would be recommended to the group of users having the same age occupation.

The user profile contains ‘genre’ of movies, the user has watched/rated in the past. According to the user profile, the movies are extracted from the dataset i.e. all the movies having at least one of those ‘genre’ as contained in user profile. Almost all the movies in the dataset have multiple genres but only the top three most watched movies ‘genre’ by the user and top 1 most watched genre (when dataset is sparse) is considered to build a user profile, according to which the new movies are recommended to the user. To improve the quality and accuracy of the recommendations, a fuzzy logic based approach is proposed.

Our proposed fuzzy algorithm uses fuzzy logic to recommend movies by predicting the ratings of the movies which are extracted from the dataset using CBF algorithm. We have developed a fuzzy recommender system that takes two features defined as ‘similarity’ and ‘dissimilarity’ as inputs and predict the movie ratings and generate movie recommendations to user with higher ratings. To further improve our recommendation results, a conformal prediction technique is employed, which only recommend items to the user with a good confidence measure. Conformal Prediction is a recent technique in machine learning and is gaining significant importance since it can be built on any existing framework of prediction or classification e.g. k-NN, SVM, decision trees, neural network, Bayesian prediction etc. [26].

Conformal prediction framework provides prediction with confidence for each classification along with region prediction (prediction of multiple classes) with different confidence levels. Predictions are associated with a measure of confidence [27]. A conformal prediction approach was initially started to solve the supervised machine learning problems. A conformal prediction works by predicting a class along with estimating the confidence levels for a given training data set [28].

Conformal prediction has two significant steps: first, computation of non-conformity measure, which is the strangeness of new object w.r.t. all the previous objects in the bag of objects. Second, take the non-conformity measure as input to get prediction region as an output. Suppose we have bag of objects o_i_ {o_1_, o_2_, o_3_…., o_n_} on which we train our classifier so it is also called a training set. Each object o_i_ is a pair of feature vector ‘a_i_’ and a class label ‘c_i_’ e.g. for object o_i =_ {a_i_,c_i_}. Now consider an unclassified object o_n+1_ with an unknown class label ‘c_y_’. The non-conformity measure is defined as the strangeness of o_n+1_ with respect to other objects in the training bag o_i_. The p-value of each class label is computed which is the confidence level of that label. The class labels having the p-value greater than the specified threshold ‘ε’ defines the prediction region of the unclassified object o_n+1_.

The extension of a conformal prediction to use with recommender system benefits the user very well as it provides recommendations of items with a level of confidence. There is not much work observed in the field of a conformal recommender system but the results guarantee its advantages in prediction used in recommender systems.

One of the main advantages of using conformal prediction algorithm is that it exhibits a validation and an exchangeability property e.g. for the validation property, if we set the confidence level at 80 percent, then there is 80 percent chance that the item being recommended is not error prone. This property of validation is proved using the data set which is identically and independently distributed (i.i.d). For the exchangeability property, consider the example of a data sequence data sequence *z*_1_,…, *z*_*l*_,…, *z*_*N*_ where *z*_*i*_ = (*x*_*i*_, *y*_*i*_) are generated in random order, if the *N*! different orderings of this sequence are equally likely, then we say that *z*_1_,…, *z*_*l*_,…, *z*_*N*_ are exchangeable [28]. A non-conformity measure [29] is denoted by ‘α’ which is calculated for each object in the bag of objects {o_1_, o_2_,…,o_n}_ and can be measured using a conformal prediction algorithm.

The major contributions of our research work are presented as follows:

1. A hybrid recommender system which solves data sparsity problem using a content based filtering approach and predicts ratings using a fuzzy based algorithm and improves the accuracy of the system by incorporating a confidence measure with each recommended item using a novel conformal prediction method forming a Hybrid Content based Fuzzy Conformal Recommender System (HCF-CRS).
2. Our proposed method works well even when the user-item matrix is sparse (when there are no ratings provided) and the user preferences (user demographic data) are unknown.
3. Our proposed recommender system is evaluated using a real dataset from MovieLens with 1M records and Movie Tweetings dataset against standard state-of-the-art RSs to prove its better accuracy and performance.

## Related work

In literature, a variety of different techniques have been used for providing recommendations to users.

For recommending items, M. Yahya [30], used demographic information from user profile. Demographic data benefited the user by overcoming the cold start problem but demographic systems does not gain much importance due to privacy and security concerns as users hesitate to provide their true demographic information. Similarly, in [31], the recommender system totally depends on the demographic data of the user and if it is not provided then the algorithm would fail to work. The use of Pearson coefficient given in the paper cannot accurately measure the correlation.

A hybrid recommendation algorithm is proposed in [32] which integrates movie feature and user interest for the similarity calculation between users, using the collaborative filtering approach for movies recommendation when user-item matrix is sparse. Experiments are performed on only one dataset of MovieLens from the year 1998 using one evaluation metric of MAE (Mean Absolute Error). Considering only MAE for evaluation of a recommender system and improving it without considering other evaluation metrics, makes the problem in some way inconsistent.

A user-based and item-based CF methods are blended together to create a switching hybrid approach [33]. A content based filtering method is also added to this hybrid approach for getting efficient and accurate recommendations. Using the MovieLens 1M dataset, the hybrid method recommends only fixed 20 movies to each user which cannot be increased.

A weighted multi-attribute based recommender system (WMARS) with user behavior analysis is proposed in [34]. It obtains implicit and explicit feedback from users and examine it. The RS has been operated on a movie web site and tested by approximately 567 users. The results for clicked movies, sequence of clicked movies, duration of watching time, rate of likes-to-dislikes are evaluated and compared only with the traditional collaborative filtering method to prove its effectiveness. The proposed approach is applied on the group of users from one city (location) only.

In [35], an importance of using confidence estimation between different recommender algorithms is discussed but no experimental results are provided [35].

A confidence estimation of the ratings prediction by collaborative filtering is used in [36], where three proposed confidence estimation algorithms are evaluated with other three confidence estimation algorithms using a confidence curve. The results are evaluated using only the confidence curves. The confidence estimation is mostly performed with CF research in literature [35, 36]. A confidence estimation approach is used with the matrix factorization CF method for recommending items using OrdRec [37] by providing confidence level of each item. Tejeda et. al. [38] proposes a recommender system for the generation of quality items such as resource materials that helps users to access relevant resource materials.

Using MovieLens dataset [39], a hybrid recommender system combines k-means and cuckoo search to achieve an improved movie recommender system. As the data is divided into several partitions and in case, if the initial partition does not work well the efficiency of the system is immensely decreased.

PCA-GAKM [40], a principle component analysis technique with genetic algorithm is proposed. A feature selection based on PCA was performed and the whole data space is transformed into a low dimension vector space. The approach performs well but cannot deal with higher dimensionality and sparsity issues from practical point of view. In [41], different ways of applying conformal prediction to matrix factorization and different non-conformity measures based on matrix factorization to evaluate the best non-conformity measure is proposed. The proposed algorithm in [41] yet failed to generate a good fraction of correct predictions and single labels at higher confidence levels.

Our proposed HCF-CRS framework, on the other hand does not require any demographic information and creates a user profile using content based filtering technique. Hence, resolving the dependency of other RS’s on user’s demographic data. A novel fuzzy based algorithm is proposed for predicting movie ratings. The proposed confidence estimation algorithm sets a threshold on the confidence level of each item which is not adopted yet in any of the discussed confidence estimation algorithms from literature and it gives better accuracy with any number of recommended movies. HCF-CRS is evaluated using the MovieLens dataset where every user has rated at least 20 movies and the Movie Tweetings dataset which is considered as the sparsest dataset where user’s ratings are mostly given to one or two movies.

## Proposed HCF-CRS framework

Hybrid Content based Fuzzy Conformal Recommender System (HCF-CRS) is focused on an effort to solve the sparsity problem and the lack of demographic data information, to predict ratings of the items and calculate the confidence level of each recommended item. The quality of recommendations provided to the user by HCF-CRS is improved by recommending only those items to the user which has confidence measure greater than the set threshold called the significance level ‘ε’.

The sparsity problem usually occurs when there are not enough ratings provided to the item by the user or when there is a new item in the database. The unavailability of user demographic data which the users feel reluctant to provide is solved by using the CBF algorithm. A fuzzy algorithm is used for the prediction of movie ratings, which are not provided by the user or there is a new movie to be recommended. A conformal prediction algorithm is incorporated with the above two defined techniques, which uses a novel non-conformity measure by taking into consideration the difference of actual and predicted ratings of an item generated by proposed fuzzy recommender system to get the confidence measurement of each recommended movie by the fuzzy system. The conformal prediction algorithm also sets a bound/threshold called the significance level ‘ε’ on the measured confidence level of the recommended items to the user and helps to further reduce the recommendation set by only recommending those items to the user with the confidence level greater than the set significance level.

Consider an example of how our proposed HCF-CRS system works on a movies dataset by learning and recommending movies to the customers according to their interests. First, using a CBF method a user profile is created by taking into consideration the movies previously watched (rated) by the users from the user history (browsing behavior). The user profile now contains the three top most watched movie ‘genre’. Thus, according to the user profile a list of movies containing all those ‘genres’ are extracted from the dataset. A fuzzy logic algorithm is applied to find the similarities and dissimilarities between the movie ‘genre’ in user profile and the selected movies from the dataset. A set of fuzzy rules are learned according to the similarities and dissimilarities score using the fuzzy logic algorithm and a rating is computed for each movie. A predicted rating is associated with each extracted movie and the highly-rated movies are recommended to the user but to make our recommendation set concise and more reliable with improved accuracy, we have built a novel conformal prediction algorithm. A non-conformal computation is performed which computes a non-conformity score between the predicted movie ratings by the fuzzy method and the actual ratings from the dataset. A non-conformity score measures how much a new item conforms to the set of already accessed items by the same user. The hybrid content based fuzzy conformal recommender system (HCF-CRS) now provides a list of movies with a confidence level, where we apply a bound on the confidence level called the significance level to recommend a set of movies to the user above the specified confidence level. This concise list of movies are the main recommended items to the user with improved accuracy.

**Learning process**. The following important parameters play the key role in the working of our proposed HCF-CRS system:

- The parameter ‘k’, which contains the top most preferred movie ‘genre’ the user has rated/watched in the past. The value of k is set at ‘3’ so we need 3 top most movie ‘genres’ that are mostly watched by the user to build a user profile. In case of sparse dataset, where the user has rated less than two items the value of ‘k’ is set to 1. The user ratings for that movie are not considered for this purpose so it can also be applied in situations when there are no ratings provided by the user but the browsing history contains the view for that movie. This parameter is learned using CBF algorithm.
- Using fuzzy logic algorithm, we define and set two features known as ‘similarity’ and ‘dissimilarity’. These features are computed using the (1) intersection of movie ‘genre’ from the user profile and each preferred movie from the dataset and the (2) difference of movie ‘genre’ in the user profile and the movie from the dataset. A set of fuzzy rules are defined and applied on the defined features, according to which the ratings are predicted for each movie for a given user. The value of the features is different for each user profile. No user ratings are considered for building these parameters and predicting ratings. A list of recommended items is generated with the highly rated predicted ratings.
- A conformal prediction approach defines and set the parameter called significance level ‘ε’. The value of confidence for each recommended movie by the fuzzy logic approach is computed which gives the confidence measure of how much a given item would be in the interest of a user. The value of confidence lies between 0 to 1 with a 1 being highly recommended item for the user. A significance level ‘ε’ is a bound-on confidence measure. We set the value of ‘ε’ as 0.45, according to which only the items above that value are recommended to the user. Hence, giving more accurate results with a concise recommendation set.

**Fig 1:**
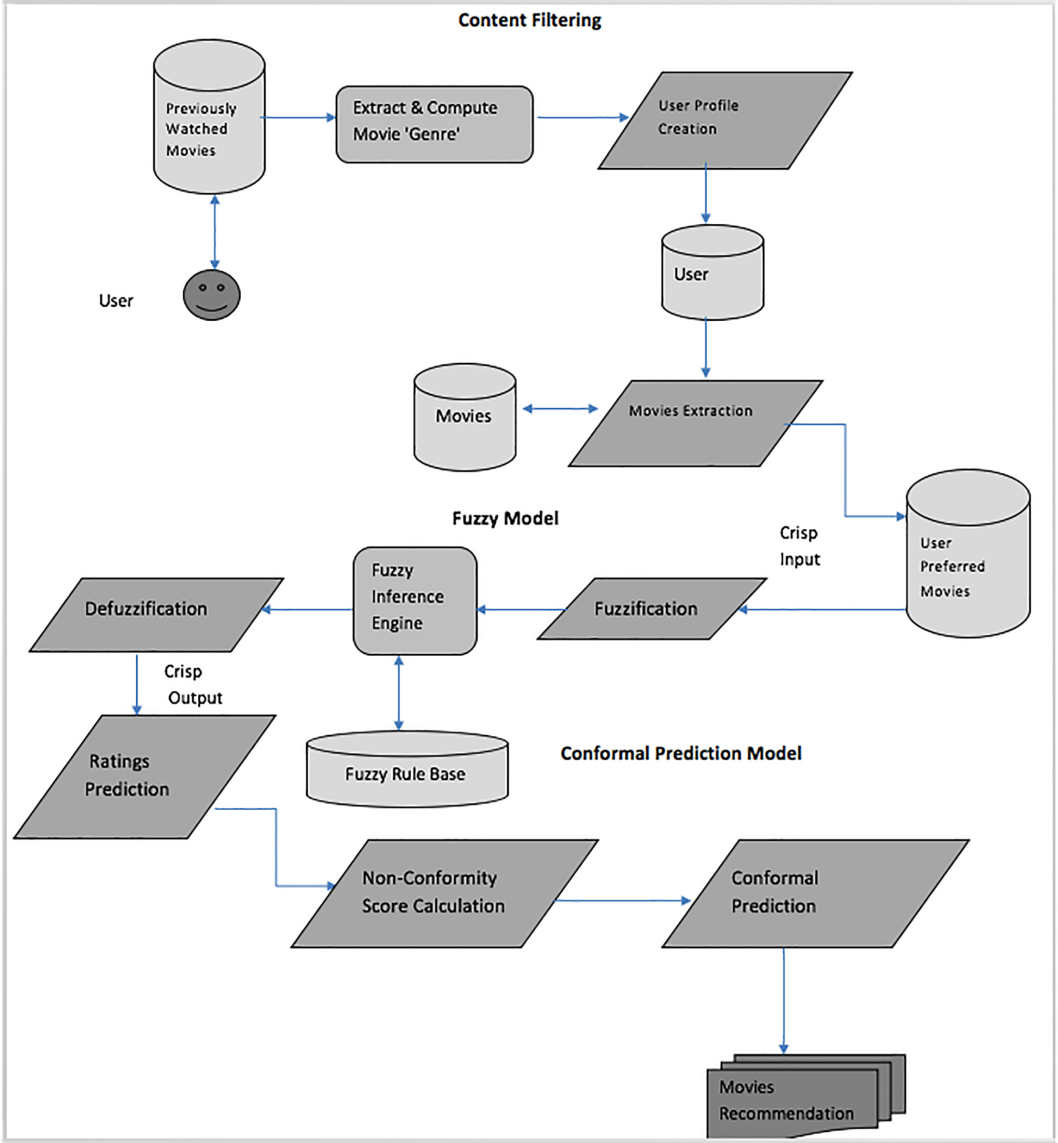
Overview of proposed HCF-CRS framework for movies recommendation.

An overview of the proposed HCF-CRS framework is given in Fig 1 and is explained in detail in the following subsections.

### CBF (content based filtering) model

The proposed Content based filtering algorithm involves the following steps with examples.

Consider a sparse dataset where there are 1 or 2 ratings provided by any user or a database with no user demographic data. Let U = {u_1_, u_2_, u_3_. . . .u_n_} be the number of users in the dataset and {r_1_, r_2_, r_3_. . . .,r_n_} be the predicted ratings on items, M = {m_1_,m_2_,m_3_. . . .m_k_} be the set of movies in the dataset. By taking into consideration the movies watched by each user in past, the ‘genre’ of each movie is recorded and the most frequently watched 'genre' of the movies is used to build up the user profile. Subsequently all the movies from the movies database containing the top K user interested 'genre' of movies are extracted.

#### User profile creation

To recommend movies to a user u_i_, we check the implicit behavior of the user i.e. the movies watched/rated by the user in the past. The ‘genre’ of each movie is recorded and the top ‘K’ most frequently watched movie 'genres' are used to create a user profile. In the proposed method, we take K = 3, to get the top 3 ‘genre’ of movies interested to the user which forms an appropriate output or K = 1 when the user-item matrix is sparse. Algorithm 1 shows the pseudo code for creating user profile.

_________________________________________________________________________

**Algorithm 1**. Creating User Profile

**Input**. User id         /*the id of user to recommend movies

**Output.** Top K 'genre' of mostly watched movies

**Load** 'genre' of each movie previously viewed/rated by the user U

**Get** frequency of each 'genre' of viewed/rated movie M

**Get** the top K most interested 'genre' for the user id U

**For** (int i = 0, i< = K, i++)

**Print** K /*user profile

end **For**

_________________________________________________________________________

#### Movies extraction

The movies are extracted from the movies database according to the user profile. The movies M from the movies database are extracted having the top ‘k’ movie ‘genre’ given in user profile. Algorithm 2 shows the pseudo code to extract movies according to the movie ‘genre’ in user profile.

_________________________________________________________________________

**Algorithm 2**. Extract movies according to the movie 'genre' in user profile

**Input**. Top K Genre of movies containing in user profile

**Output**. M /* movie ids

**Load** user profile

**if** (genre.userProfile==genre.movieDB) **then**

extract movie

end **if**

_________________________________________________________________________

**Fig 2:**
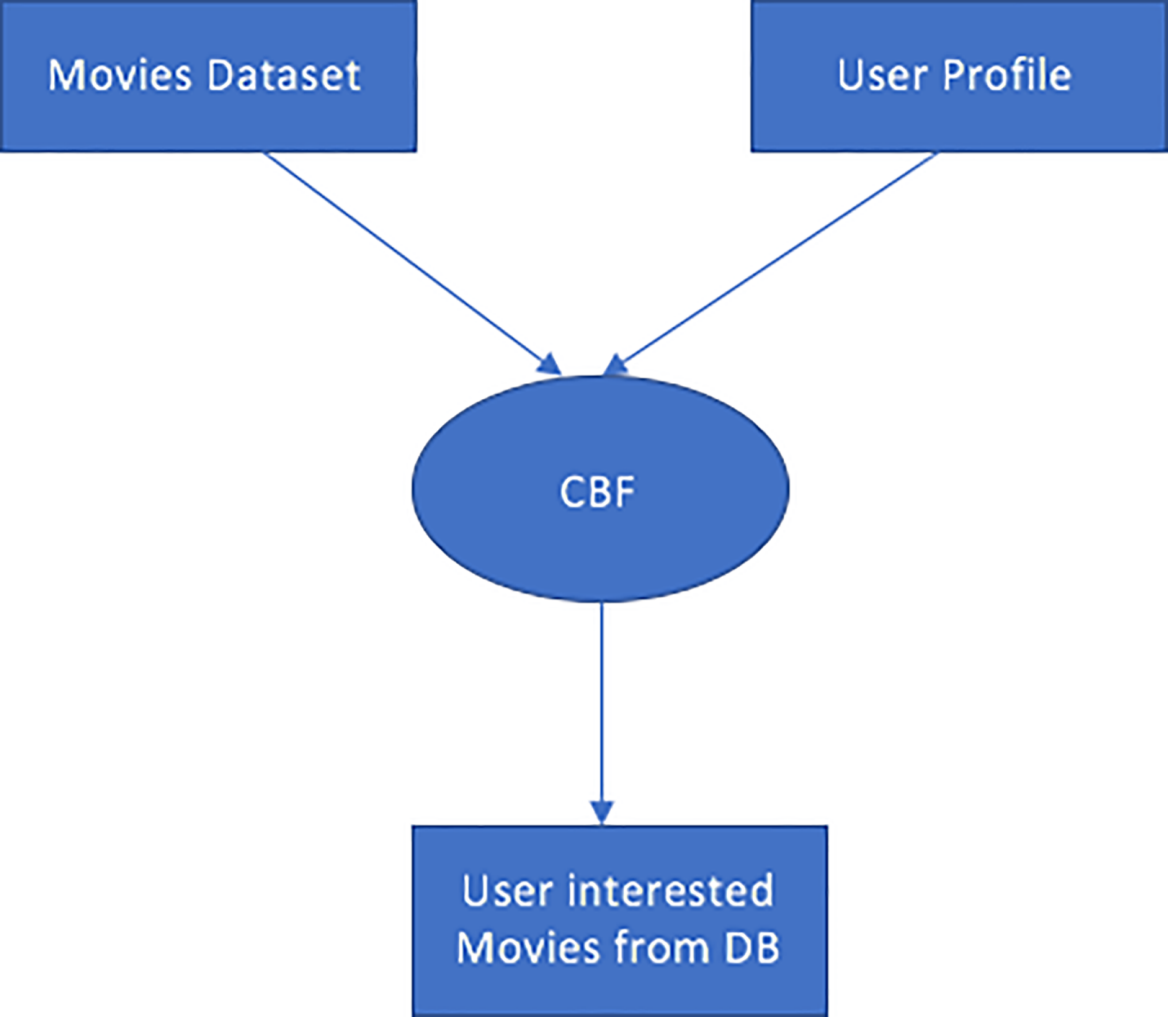
CBF algorithm.

Fig 2. illustrates the CBF algorithm. The algorithm is applied on movies dataset and the user profile to extract a list of user preferred movies from the dataset.

### Fuzzy model

A fuzzy algorithm is proposed that is used to find the similarity and dissimilarity between the movie ‘genre’ in user profile and the movie ‘genre’ in extracted movies from database. The fuzzy system considers ‘similarity’ and ‘dissimilarity’ as two features and take them as crisp input to the Fuzzy Inference System (FIS).

The input features are fuzzified by the FIS. The FIS extracts the rule set from the knowledge base and generates predicted rating as output for each movie.

To associate an FIS to CBF, we have used two input variables, one is ‘similarity’ and the other is ‘dissimilarity’. ‘Similarity’ is defined with three membership functions; ‘low’, ‘medium’ and ‘high’, while ‘dissimilarity’ is defined with membership functions called ‘no’, ‘low’ and ‘high’.

The ‘similarity’ feature is calculated by finding the intersection of movie ‘genre’ between the user profile and each extracted movie by the CBF algorithm. While the ‘dissimilarity’ feature is computed as the total number of movie ‘genre’ present in the extracted movie but not in the user profile.

For example, A user profile contains three most watched ‘genre’ of movies e.g. ‘Animation’, ‘Comedy’ and ‘Action’ while the extracted movie from movies database contains ‘genre’ e.g. ‘Comedy’ and ‘Drama’. The ‘similarity’ feature computed here is 1 and the ‘dissimilarity’ feature is also 1. Eq 1 and Eq 2 are used for computing the ‘similarity’ and ‘dissimilarity’ features.

$${\mathrm{Simm}=[\mathrm{Genre}}_{\mathrm{userprofile}}{\cap \mathrm{Genre}}_{\mathrm{movie}]}$$ (1)

$$\mathrm{Dissimm}=\left[\sum \left({\mathrm{Genre}}_{\mathrm{userprofile}},{\mathrm{Genre}}_{\mathrm{movie}}\right)\right]‑\;\mathrm{Simm}*2$$ (2)

The Eq (1) for computing similarity takes the intersection of movie genre present in the user profile and in the movie extracted from the dataset i.e. the number of matched movie ‘genre’. Eq (2) calculates the dissimilarity feature by taking the sum of all the genres from the user profile and the extracted movie and subtracting it from 2 times the similarity feature.

#### FIS (fuzzy inference system) algorithm

FIS takes two input variables ‘similarity’ and ‘dissimilarity’ as crisp inputs which are further fuzzified to get the output ‘Predicted Rating’. These two steps of FIS algorithm are divided into two algorithms.

Algorithm 3 shows the pseudo code for the computation of ‘similarity’ and ‘dissimilarity’ features and algorithm 4 shows the pseudo code to predict ratings for movies using the ‘similarity’ and ‘dissimilarity’ features. The FIS evaluates the input variables and apply fuzzy inference rules from the rule base. The output is defuzzified to get the predicted ratings of movies.

_________________________________________________________________________

**Algorithm 3**. Computing ‘similarity’ and ‘dissimilarity’ features

**Input**. User profile and movies extracted from CBF algorithn /*

**Output.** ‘Similary’ and ‘dissimilarity’ score

**Load** user profile and movies /* movies extracted by CBF algorithm

**Compute** Simm = [Genre_userprofile_ ∩ Genre_movie_]

**Compute** Dissimm = [∑(Genre_userprofile_,Genre_movie_)]-Simm*2

**Get** Score

_________________________________________________________________________

_________________________________________________________________________

**Algorithm 4**. Predict ratings for Movies using FIS

**Input**. Similarity and Dissimilarity score /* getting the difference of movie genre in user profile & extracted movies by CBF

**Output.** Predicted Rating R of movies /* rating assigned to extracted movie

**Evaluate** FIS with input variables

**Apply** Fuzzy Inference Rules

**Get** defuzzified output ‘Predicted Ratings’

**Display** Top rated Movies for recommendation

_________________________________________________________________________

**Fig 3:**
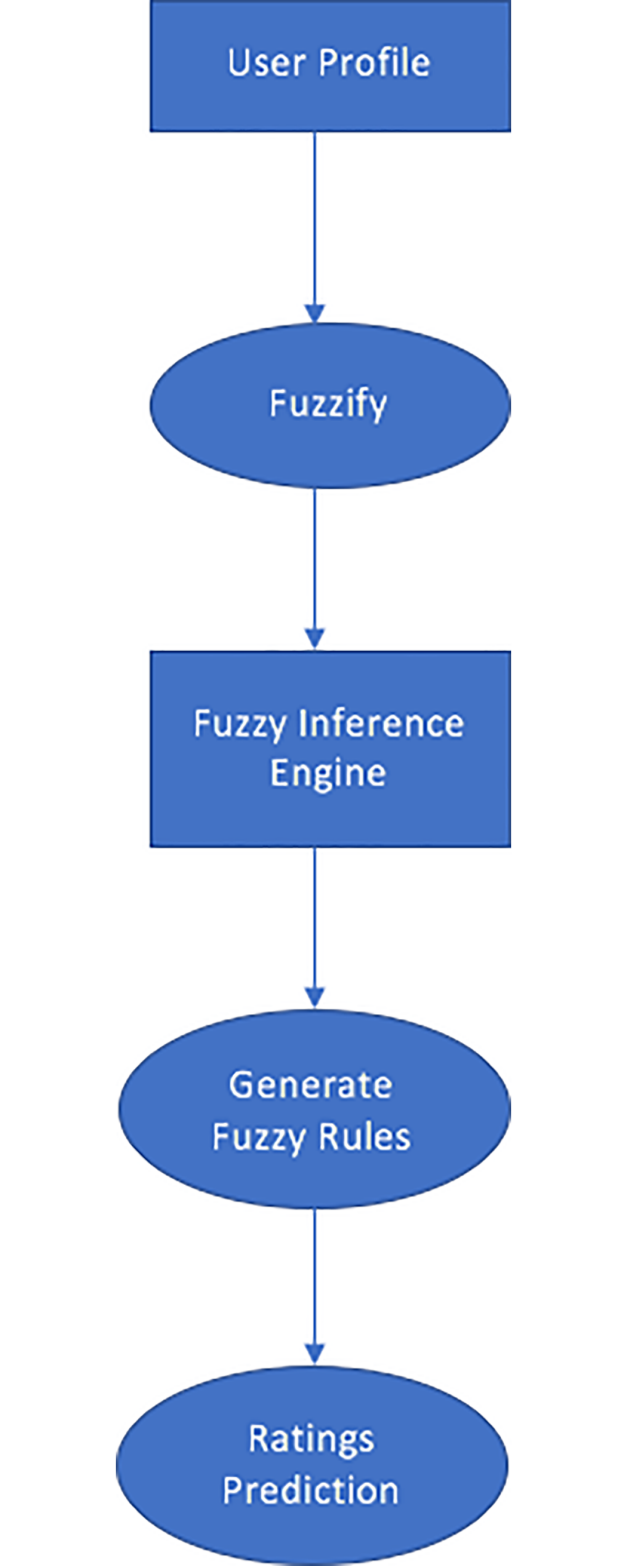
Fuzzy algorithm.

Fig 3. illustrates the Fuzzy algorithm. The input features from the user profile are extracted and fuzzified to fed into a fuzzy inference system which applies fuzzy rules on them to get the output as predicted rating.

#### Fuzzy model implementation

The proposed Fuzzy Recommender System is implemented using jfuzzylogic.

***Jfuzzylogic***

Jfuzzylogic is an open source fuzzy logic library that is written in java and it assists in fuzzy system developments. We have used jfuzzylogic in the implementation of our proposed fuzzy system [42]. A Fuzzy Control Language (FCL) is used which is a standard language for programming fuzzy systems. FCL is a language defined in the IEC 61131 part 7 specification [43].

**Fuzzy Control Language (FCL)**

As the name indicates FCL is a control language. Therefore, the control block is the main concept which contains the input and output variables. The program is executed in parallel as there is no specified execution order.

In our research work we have used FCL and defined it in the following way:

A fuzzy inference system contains one or more function blocks. The function block is defined first. The input and output variables are defined afterwards, called the ‘similarity’ and ‘dissimilarity’ features. We define a FUZZIFY block, which contains the input features e.g. ‘similarity’ and ‘dissimilarity’. The block contains one or more terms called the linguistic terms. Each term has a name and a membership function. In the given case, the input variable ‘similarity’ has three terms defined as {Low, Medium, High} with membership values {1, 2, 3}. Similarly, the input variable ‘dissimilarity’ is fuzzified into three terms {No, Low, High} with values {(0,1), 2, 3}. When the membership function of ‘similarity’ equals to 1, it means that the number of movie ‘genre’ in user profile matches with only one ‘genre’ in extracted movies therefore, the output ‘similarity’ function is defined as ‘Low’. Similarly, if the ‘similarity’ of movie ‘genre’ in user profile matches with two ‘genre’ of movies in extracted movies then the output similarity function is defined as ‘Medium’ and finally if the ‘similarity’ of movie ‘genre’ in user profile matches with three or more ‘genres’ in extracted movies then the output similarity function is defined as ‘High’.

The membership function of ‘dissimilarity’ with ‘No’ occurs when there is no or only one movie ‘genre’ in user profile which is not matched with the ‘genre’ of movies in the extracted movie from the dataset. If the ‘dissimilarity’ of a movie is ‘two’, it means there are two ‘genre’ of the extracted movie that are not present in the user profile then the membership function is defined as ‘Low’. Similarly, if the ‘dissimilarity’ of a movie is ‘three’ i.e. there are three ‘genre’ of the extracted movie that are not present in the user profile then the membership function is defined as ‘High’.

**Fig 4:**
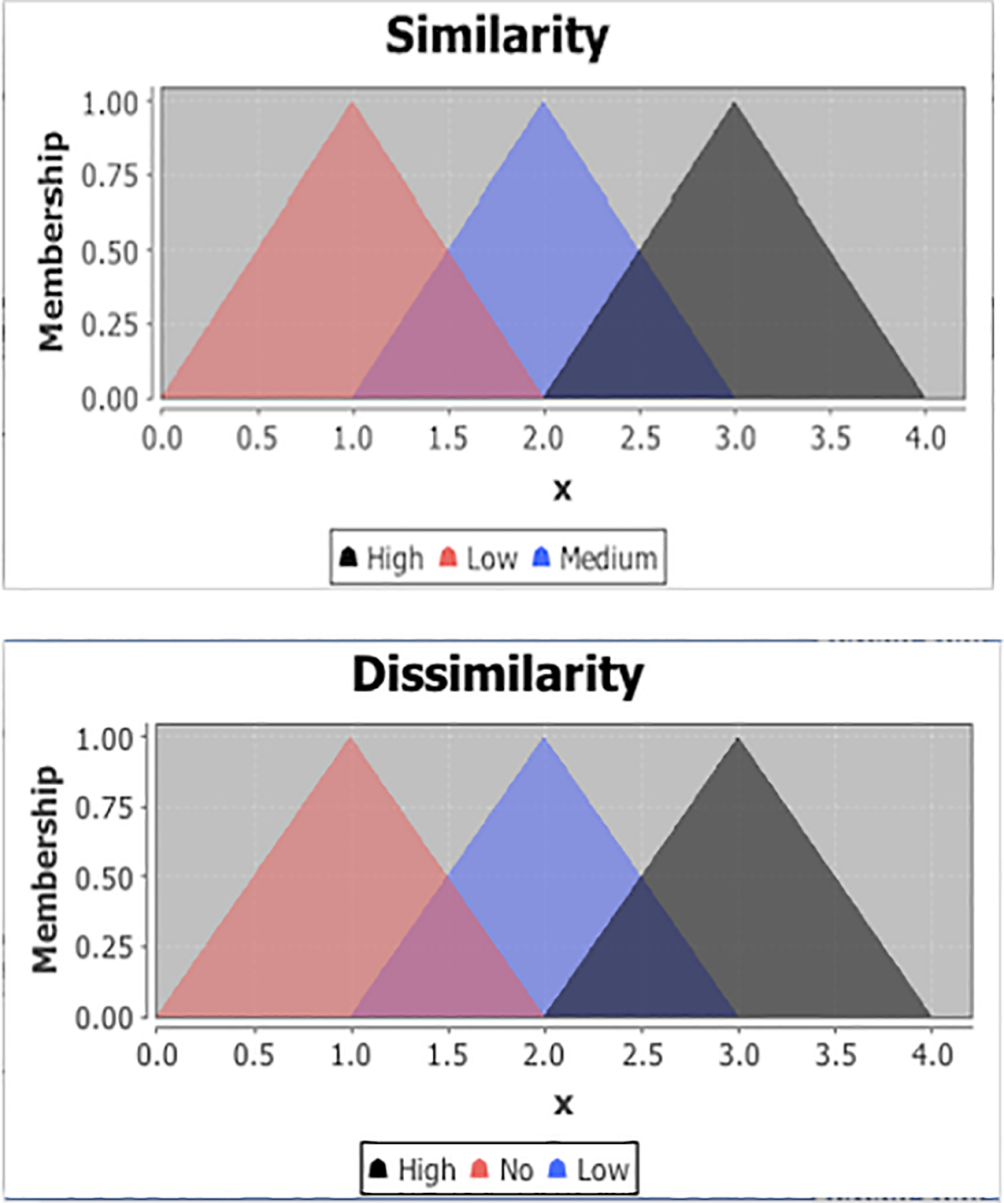
Membership function for input variable ‘similarity’ and ‘dissimilarity’.

The corresponding graphs of the ‘similarity’ and ‘dissimilarity’ features are illustrated in Fig 4(A) and 4(B).

The output variable known as the ‘predicted rating’ is defuzzified to get a ‘real’ output. The output to be defuzzified is defined with three linguistic terms {Poor, Good, Excellent}. The values of the defined terms shows how the membership values of ‘similarity’ and ‘dissimilarity’ leads to the predicted rating to be ‘Poor’, ‘Good’ or ‘Excellent’.

**Fig 5:**
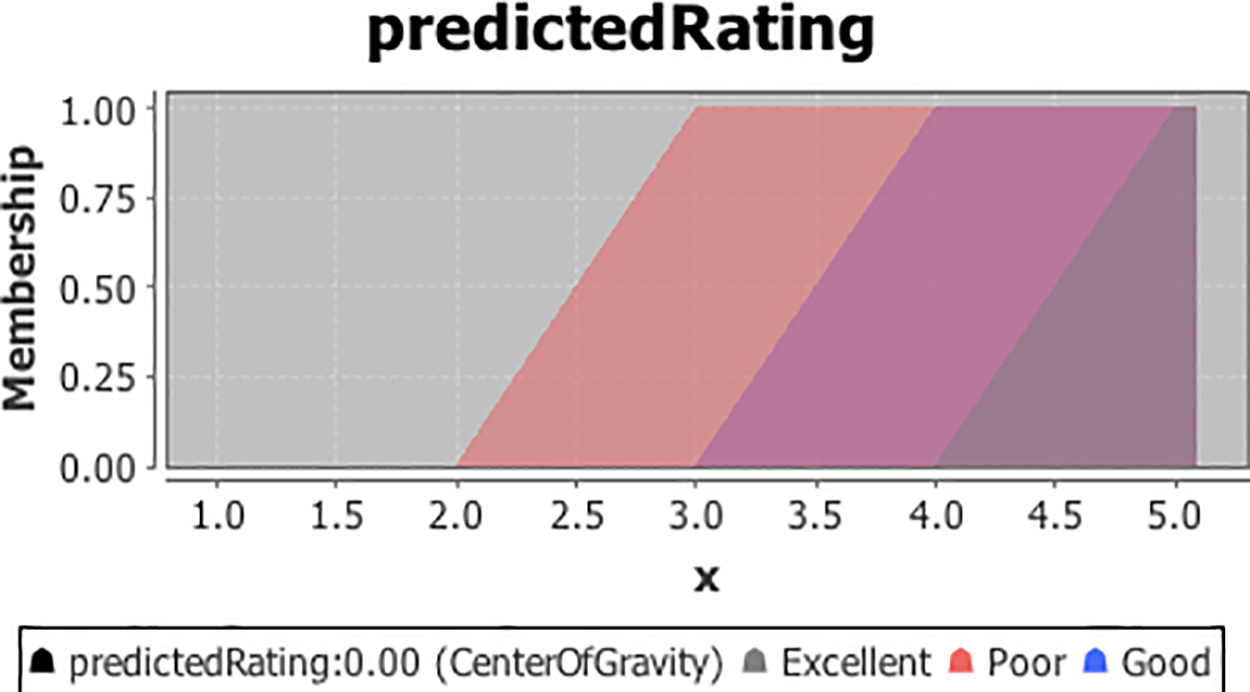
Membership function for the output variable ‘predicted rating’.

Next, we have used ‘center of gravity’ as defuzzifier method and the default value is set to zero which states that no rule activates the defuzzfier. The membership functions of ‘predicted rating’ as ‘Poor’, ‘Good’ and ‘Excellent’ is illustrated in graph given in Fig 5.

A Mamdani defuzzification system is used to apply the fuzzy inference rules defined in Table 1 and get the output. In real world, the measurements and actions are expressed as crisp input therefore a Mamdani Defuzzification system is applied to fuzzify crisp inputs to get the fuzzy inputs and defuzzify the fuzzy outputs to get the crisp outputs [44].

**Table 1:** Fuzzy inference rules.

**Dissimilarity**	**Similarity**			
		**Low**	**Medium**	**High**
	**No**	Good	Good	Excellent
	**Low**	Good	Good	Excellent
	**High**	Poor	Poor	Good

Table 1 defines the fuzzy rules that are applied on the input features, ‘similarity’ and ‘dissimilarity’ and are obtained from the user preferred movie list.

### Conformal prediction model

In this section, we have proposed our conformal prediction algorithm which is built upon our proposed content based filtering algorithm and the fuzzy logic algorithm. A non-conformity measurement and p-value calculation is performed to get the confidence measure of each recommended item to get awareness of how much a certain item is important to them as well as by setting a bound/threshold on the measured confidence level called a significance level ‘ε’ we can restrict only certain items above that bound to be recommended to user. Hence, making the recommendation set concise and accurate. In literature, most researchers using the collaborative filtering techniques [45, 46] and the matrix factorization techniques [47, 48] for recommending items using confidence measures, have associated the confidence measures with the ratings of the items e.g. if there are 5 possible ratings for an item, there are 5 different confidence measures associated with each item. This results in a complexed prediction region. The users are usually more interested in getting the confidence measures for an item recommended to them. Our proposed Hybrid Content-based Fuzzy Conformal Recommender System (HCF-CRS) helps to determine the prediction region according to the set significance level ‘ε’. The two important parts of a conformal prediction algorithm is the non-conformity measurement and the p-value calculation to compute the confidence of each item to be recommended.

**Non-conformity measure** is the fundamental part of any conformal based system. In different domains of conformal prediction, a different non-conformity measure is applied.

A non-conformity measure is mainly used for determining how much the new item conforms to the set of already accessed items. In our proposed CRS, the non-conformity measurement is computed as the difference of ratings provided by the user and the predicted ratings estimated by the proposed fuzzy algorithm. The set of predicted ratings by proposed fuzzy algorithm is denoted by P_i_ = {P_1_, P_2_, P_3_……P_n_}, where P_n+1_ is the predicted rating for the new item for a given user. For our proposed non-conformity measure, a bag of training data O_i_ = {O_1_, O_2_, O_3_……O_n_} is the set of movies watched and rated by the user U_i_ is considered with the set of ratings provided by the user as R_i_ = {R_1_, R_2_, R_3_……R_n_}. A new item for recommendation is denoted by O_n+1_. The main purpose of the non-conformity measure is to check how well the new item O_n+1_ conforms to the set of utilized objects O_i_. The proposed non-conformity measure of all the items in the training set for a given user U_i_ is calculated as follows:

$${\mathrm{Score}}_{\left(\mathrm{non}‑\mathrm{confirmity}\right)}O_{i}=\left|{\mathrm{predictedRating}}_{\left(\mathrm{Fuzzy}\right)}-{\mathrm{actualRating}}_{\left(\mathrm{User}\right)}\right|$$

Or

$${\mathrm{Score}}^{\mathrm{Oi}}=\left|P_{i}‑R_{i}\right|$$ (3)

According to the proposed non-conformity score, Eq (3) computes the difference of user rating given to the item and the predicted rating computed by the proposed fuzzy model for each item in the training set O_i_. The non-conformity score of the new item whose rating is not provided by the user is computed as:

$${\mathrm{Score}}_{\left(\mathrm{non}‑\mathrm{confirmity}\right)}O_{n+1}=\left|{\mathrm{predictedRating}}_{\left(\mathrm{Fuzzy}\right)}‑\;{\mathrm{MovieRating}}_{\left(\mathrm{average}\right)}\right|$$

Or

$${\mathrm{Score}}^{\mathrm{On}+1}=\left|P_{n+1}‑{\mathrm{AvgM}}_{i}\right|$$ (4)

According to the Eq (4), the difference of the predicted rating for the new item (whose actual rating is not given by the user) and the average rating for that movie by all other users is considered to compute the non-conformity score. To validate the proposed non-conformity score computation, consider an example for non-conformity score calculation. Given a dataset O_i_ = {O_1_, O_2_, O_3_……O_n_} of rated movies with the set of ratings R_i_ = {R_1_, R_2_, R_3_……R_n_} by a set of users U_i_ = {U_1_,U_2_,U_3_,…U_n_} and a set of movies with predicted ratings P_i_ = {P_1_, P_2_, P_3_…..P_n,_ P_n+1_ } by proposed fuzzy model. The non-conformity measure {α_1_,α_2_, α_3_….,α_n_} is computed for any user as follows:

$$\begin{array}{l} \alpha_{1}={\mathrm{Score}}^{O1}\;\left(R_{1},P_{1}\right)=\left|P_{1}‑R_{1}\right|\\ \alpha_{2}={\mathrm{Score}}^{O2}\;\left(R_{2},P_{2}\right)=\left|P_{2}‑R_{2}\right|\\ \alpha_{3}={\mathrm{Score}}^{O3}\;\left(R_{3},P_{3}\right)=\left|P_{3}‑R_{3}\right|\\ .\\ .\\ \alpha_{n}={\mathrm{Score}}^{\mathrm{On}}\;\left(R_{n},P_{n}\right)=\left|P_{n}‑R_{n}\right| \end{array}$$

For recommending a new item O_n+1_ to the user from dataset, the non-conformity score α_n+1_ computation is performed using average rating AvgM_i_ for that movie which is computed from the dataset by taking average of all ratings given by all the users for that movie.

$$a_{n+1}={\mathrm{Score}}^{\mathrm{On}+1}\left(R_{n+1},\;P_{n+1}\right)=\left|P_{n+1}‑{\mathrm{AvgM}}_{i}\right|$$ (5)

The proposed recommender system satisfies the exchangeability property of conformal prediction as the order of objects from bag of training samples is irrelevant. For example, a set is considered identically, and exchangeability distributed when, {1, 2, 3} = {2, 3, 1} = {3,1,2}. This property of exchangeability is fulfilled using the non-conformity measure above.

#### P-value

The p-value is computed using the non-conformity measure. The p-value for a new item O_n+1_ with respect to the predicted rating P_n+1_, computed from the fuzzy recommender system is defined as follows.

$$p\left(O_{n+1}\right)=\left|\{i\right|1\leq i\leq n+1,\;{\mathrm{Score}}^{\mathrm{On}}\left(R_{n},P_{n}\right)\geq {\mathrm{Score}}^{\mathrm{On}+1}\left(R_{n+1},P_{n+1}\right)\}|/n+1$$ (6)

Eq (6) computes the p-value of the item by selecting only those movies which have non-conformity score greater than or equal to the non-conformity score of the new movie to be recommended and is divided by the total number of movies rated by that user U_i_ plus 1.

The movie is recommended to the user if p(O_n+1_) is greater than the significance level ε such as p(O_n+1_) > ε. The items with the p-values greater than the defined significance level are considered under prediction region Γε. The items having greater p-values are finally recommended to the user. The p-value represents the confidence level of the recommended item from the proposed conformal recommender system. Algorithm 5 demonstrates the algorithm for getting recommendation set using conformal prediction.

_________________________________________________________________________

**Algorithm 5**. Getting recommendation set using Conformal Prediction

**Input**. User U_i_

**Output**. Recommendation set ⎡^ε^ with confidence values

**for** each given user

  Compute *Score*^*Oi*^ and Score^On+1^ using equation

**end**

    Compute p(O_n+1_) using equation         /* p_value

    **If** p(O_n+1_) > εthen ⎡^ε^ to U_i_         /*bound on confidence level

**End**

_________________________________________________________________________

By implementing the conformal prediction algorithm over the content-based fuzzy recommender system, the recommendation set is eventually reduced, giving precise set of recommendations to the user with improved accuracy. A smaller recommendation set is also more informative, relaiable and efficient.

**Fig 6:**
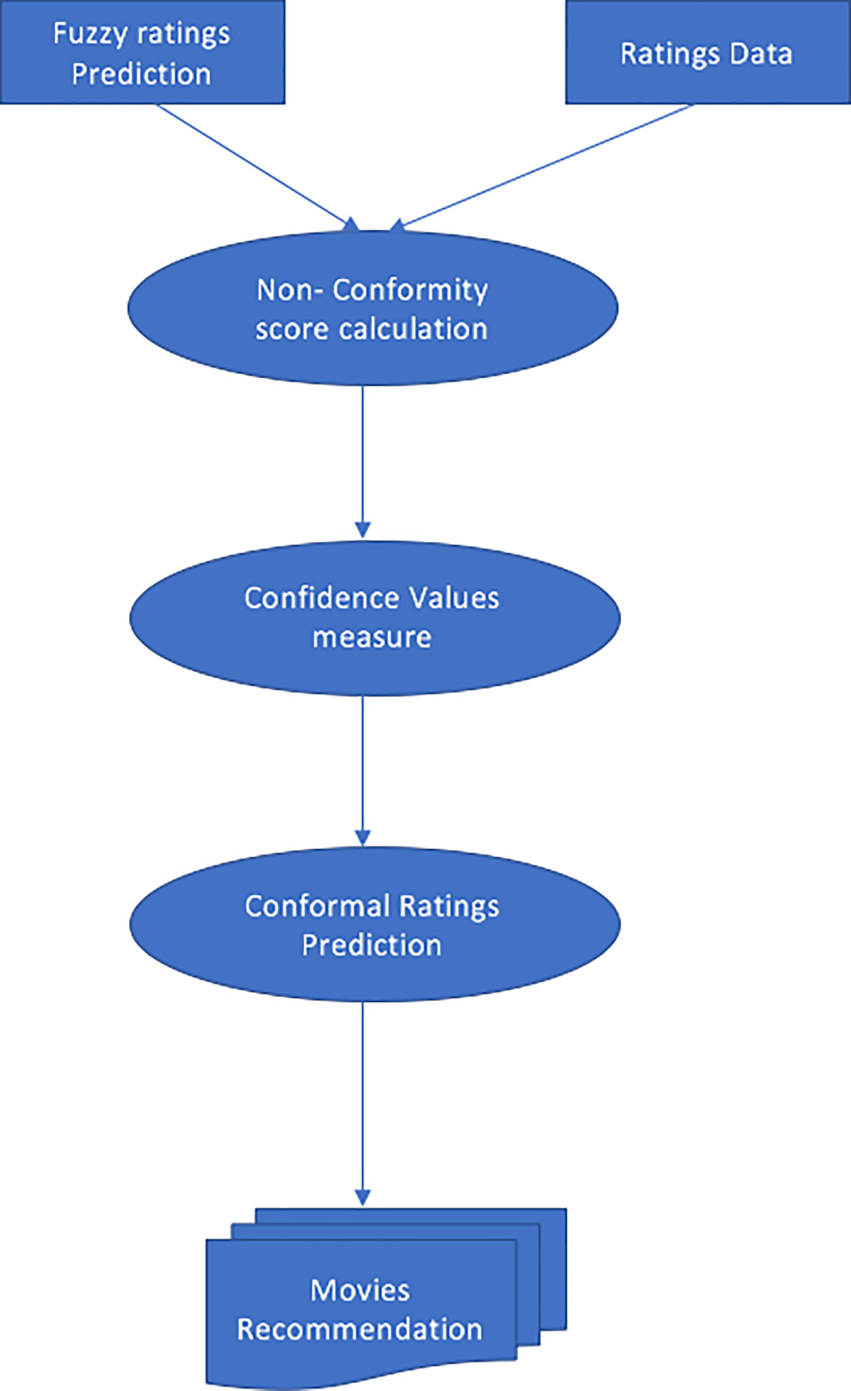
Conformal prediction algorithm.

Fig 6. depicts the conformal prediction algorithm. A non-conformity score calculation requires ratings from the dataset and the predicted ratings from the fuzzy system to compute the conformal ratings prediction for movies recommendations.

#### Conformal prediction example

To understand the concept of conformal prediction, consider an example computing non-conformity measure based on k-nearest neighbors (KNN) [49]. Suppose our example is composed of one training set containing six objects e.g. O_i_ = {o_1_, o_2_, o_3_, o_4_, o_5_, o_6_} and three class labels {1, 2, 3} using 1NN algorithm. The training set is extended into the following elements: attribute vector and class label {(a1, c1), (a2, c2), (a3, c3), (a4, c4), (a5, c5), (a6, c6)} = {(0.9, 1), (1.2, 1), (1.9, 2), (2.2, 2), (2.7, 3), (3.1, 3)}. Now consider an unclassified object o7 = (a7, c7) = (2.4, ?). We will predict the class label for the object o6 using the KNN and by allocating each available class label to c6 such as c6 = {1, 2, 3}.

For **c7 = 1**, o7 becomes (2.4, 1). now appending this set to the training set we get the following set: {(0.9, 1), (1.2, 1), (1.9, 2), (2.2, 2), **(2.4, 1)**, (2.7, 3), (3.1, 3)}. By applying the 1NN algorithm we can get the class label prediction for each of the attributes in the training set. For example, the class label for a1 is predicted as 1 according to the KNN algorithm and the actual class label is also 1. Therefore, the non-conformity measure is α1 = (1–1) = 0. Similarly, the non-conformity measure for other objects in the training set is α2 = 0, α3 = 0, α4 = 1, α5 = 2, α6 = 0. The non-conformity measure for the new unclassified object is as the nearest neighbor for new object is a4 whose class label is 2, so α7 = (2–1) = 1. The p-value for the class label c7 = 1, is computed using the equation defined in section 3.2. i.e.

$$P\left(7,\;1\right)=\#\left\{\alpha_{i}\in A|\alpha_{i}>=\alpha_{k}\right\}/\#A$$

P(7,1)=3/7=0.42

Similarly, for c7 = 2, o7 becomes (2.4, 2). now appending this set to the training set we get the following set: {(0.9, 1), (1.2, 1), (1.9, 2), (2.2, 2), **(2.4, 2)**, (2.7, 3), (3.1, 3). The non-conformity measures for all the objects in training set are: α1 = 0, α2 = 0, α3 = 0, α4 = 0, α5 = 1, α6 = 0 and α7 = 0. The p-value for the class label c7 = 2 is computed as:

$$P\left(7,\;2\right)=\#\left\{\alpha_{i}\in A|\alpha_{i}>=\alpha_{k}\right\}/\#A$$

P(7,1)=7/7=1

For c7 = 3, o7 becomes (2.4, 3). Appending this set to the training set we get the following set: {(0.9, 1), (1.2, 1), (1.9, 2), (2.2, 2), **(2.4, 3)**, (2.7, 3), (3.1, 3). The non-conformity measures for all the objects in training set using 1NN are: α1 = 0, α2 = 0, α3 = 0, α4 = 1, α5 = 0, α6 = 0 and α7 = 1. The p-value for the class label c7 = 2 is computed as:

$$P\left(7,\;3\right)=\#\left\{\alpha_{i}\in A|\alpha_{i}>=\alpha_{k}\right\}/\#A$$

P(7,1)=2/7=0.285

If the threshold or significance level ε is set as ε = 0, the prediction region according to the p-value we get is all the class labels having value greater than 0 i.e. Γ^0^ = {1, 2, 3} with 100% confidence. For setting ε = 0.3, the prediction region is the p-values above 0.3 i.e. Γ^0.3^ = {1, 2} and the confidence level for both class labels is (1–0.3) = 0.7. For significance level ε = 0.5, Γ^0.5^ = {2} with the confidence measure of 0.5.

The proposed conformal prediction algorithm works best when there are many movies rated/watched by the user as the conformal framework improves the quality of recommendations generated by the content-based fuzzy algorithm by making them concise. The MovieLens dataset works best in such situations. On the other hand, the Movie Tweetings dataset is very sparse so the fuzzy framework gives us slightly better results than by using our content based fuzzy framework.

## Experimental evaluation and results

In this section, the experimental evaluation of our proposed HCF-CRS is carried out.

All the algorithms are implemented in Java programming language.

### Real datasets

We tested our algorithms on two datasets, MovieLens 1M (http://grouplens.org/datasets/movielens) and Movie Tweetings (https://github.com/sidooms/MovieTweetings).

#### MovieLens 1M dataset

We have used MovieLens 1M dataset [50] containing 1 million ratings from users. MovieLens 1M is a real dataset containing 1,000,209 anonymous ratings of approximately 3,900 movies made by 6,040 MovieLens users. This Dataset is one of the publically available datasets collected by the University of Minnesota and is associated with their online movie recommendation system. The dataset has been widely used for offline experimental evaluation of recommender systems. The dataset contains User IDs that range between 1 and 6040, the Movie IDs range between 1 and 3952 and the ratings are made on a 5-star scale.

#### Movie tweetings dataset

The dataset is transformed in the same format as of MovieLens so performing the evaluation. Movie Tweetings dataset consists of ratings of the movies collected from the tweets on Twitter. Unlike MovieLens dataset where the user has rated at least 20 movies, this dataset exhibits extreme sparsity as some users have only rated just 1 item but this dataset incorporates ratings on the newest and most relevant movies [51]. The dataset contains nearly 7000 ratings from users.

Using the movie tweetings dataset, we can understand which algorithm works best on different kinds of users.

The data format for both datasets: MovieLens and Movie Tweetings are identical but the only difference being is the data sparsity issue, with most of the users providing rating to only 1 or 2 movies. In such case, the recommendations are generated precisely but the evaluation for sparse users cannot be done precisely.

### Evaluation metrics

The following evaluation metrics are used for measuring the accuracy and performance of our proposed HCF-CRS framework in comparison to other methods:

**MAE**. Mean Absolute Error is the most widely used metric by the research community. The MAE is calculated using the equation given in (7) [52]:

$$MAE=1/n\;{\sum {}^{n}}_{k=0}(|e_{k}‑e_{j}|)$$ (7)

In our work, MAE is calculated by taking the absolute difference of the predicted rating and the actual rating and dividing it by the total number of entries.

**Precision.** is defined as a measure of exactness [53] and it determines the number of relevant items retrieved out of all items that are retrieved e.g. from the movies dataset, precision is the number of recommended movies that are good. In our work, precision this calculated dividing the good movies recommended with all the recommendations given. The formula deduced for precision is given in Eq (8).

Precision=TP/(TP+FP) (8)

**Recall.** is defined as a measure of completeness [53] and it determines the number of relevant items retrieved out of all relevant items e.g. in case of movies dataset recall is defined as the number of all good movies recommended. In our work, recall is calculated by dividing the good movies recommended with all the good movies. The formula deduced for recall is given in Eq (9).

Recall=TP/(TP+FN) (9)

To understand the formulae of precision and recall, consider the confusion matrix in Table 2 which is given to explain the notation given in Eqs (8) and (9). Precision and Recall are used to determine the accuracy of the system. Precision and Recall somewhat complement each other as when we want to increase precision, the recall decreases automatically [53]. It is quite cumbersome to compare our results of precision and recall with other systems so we would compute a single measure from it called an F-measure. To get a more balanced view of performance of our system an F-measure is used.

**Table 2:** Confusion matrix.

	ACTUAL RATING		
PREDICTED RATING		Actual Good	Actual Bad
	Predicted Good	True Positive (TP)	False Positive (FP)
	Predicted Bad	False Negative (FN)	True Negative (TN)

**F-measure.** is the harmonic mean of precision and recall and one of the most commonly used measures in machine learning and information retrieval [54]. Eq (10) gives the formulae of f-measure for comparison.

F=2*(precision*recall)/(precision+recall) (10)

An explicit way to present the prediction results of the recommender system is to use a confusion matrix given in Table 2 for measuring precision and recall values. According to the confusion matrix, there is a true positive when the actual and predicted ratings are the same. We have set the threshold of good rating at 3 and the items are considered good whose rating is 3 or above. A false positive occurs when the predicted rating is good but the true rating is bad which means that the rating is less then 3. A false negative occurs when the actual rating is 3 or above but the predicted one is less then 3. Similarly, a true negative is hit when the actual and predicted ratings both are less then 3 and equal.

### Experimental design

We have used the MovieLens 1M dataset and the Movie Tweetings dataset for evaluating our proposed recommender system: HCF-CRS. In MovieLens dataset, the users are divided into five folds by picking users randomly from the dataset. Each fold contains 10–15 users which we have selected randomly from the dataset. In Movie Tweetings dataset, where most of the users have only rated 1 or 2 movies, the evaluation is performed by dividing the dataset into set of users who have rated less than 3 items and the set of users who have rated 3 or more items to test the effectiveness of our proposed recommender system. In this way, the users are divided into 4 folds. With each of 2 folds having 10–15 users who have rated less then 3 items and the other 2 folds having same number of users who have rated 3 or more then 3 items.

For evaluation, 90% of the dataset is used for training and the remaining 10% is used for testing. The proposed system is trained at runtime on the given dataset. Different folds of users are formed by randomly picking different number of users from dataset and calculating their MAE, Precision, Recall and F-measure.

The experiment is designed as follows:

1. The proposed isolated content based filtering algorithm generates a list of movies from the dataset which contains the same movie ‘genre’ as in user profile.
2. Using a hybrid content based fuzzy RS, each ‘genre’ of the extracted movie is matched with the every ‘genre’ in user profile and ‘similarity’ and ‘dissimilarity’ features are computed.
3. The ‘similarity’ and ‘dissimilarity’ features are given as input to the fuzzy inference system where fuzzy rules are applied to them.
4. According to the fuzzy rules, ratings are predicted for each movie.
5. The highly-rated movies are then extracted to be recommended to the user.
6. To improve the quality of recommendations and to make the recommendation set concise, a novel non-conformity computation is performed on the predicted ratings, a confidence level is measured for each recommended item and a bound ε is set on the confidence value so that only the movies with good confidence level is added to the recommendation set.
7. To test the accuracy of our proposed HCF-CRS, an MAE calculation is performed along with precision, recall and f-measure on the recommendations generated. These evaluation metrics calculate the mean difference of predicted ratings and the actual ratings along with the confidence value of each recommended item.
8. The proposed HCF-CRS is compared with the results obtained from our proposed CBF algorithm, the fuzzy model and other state-of-the-art recommender systems.

### Experimental results

In this section, an experimental evaluation of our proposed CBF algorithm, the fuzzy algorithm and the hybrid framework HCF-CRS is performed on real datasets by calculating their MAE, precision, recall and f-measure and the results are compared with our proposed isolated techniques as well as other recommender systems from literature using the same datasets and evaluation metrics.

We have integrated three different techniques to implement our framework HCF-CRS to overcome the shortcomings of individual ones. First, we have evaluated the results by applying only the CBF algorithm and observed that the system gives better results as compared to few recent state of the art proposed RS’s. Second, we have tested our proposed fuzzy algorithm and observed that it performs better by giving good quality recommendations with better performance as compared to when using an isolated CBF approach. By applying a conformal prediction technique on the fuzzy system and forming a hybrid framework, the recommendations are provided with a set of confidence level and the recommendation set is reduced while providing the recommendation of items within the set threshold (with confidence level). The quality and accuracy of recommendation is significantly improved as illustrated in the results.

**Fig 7:**
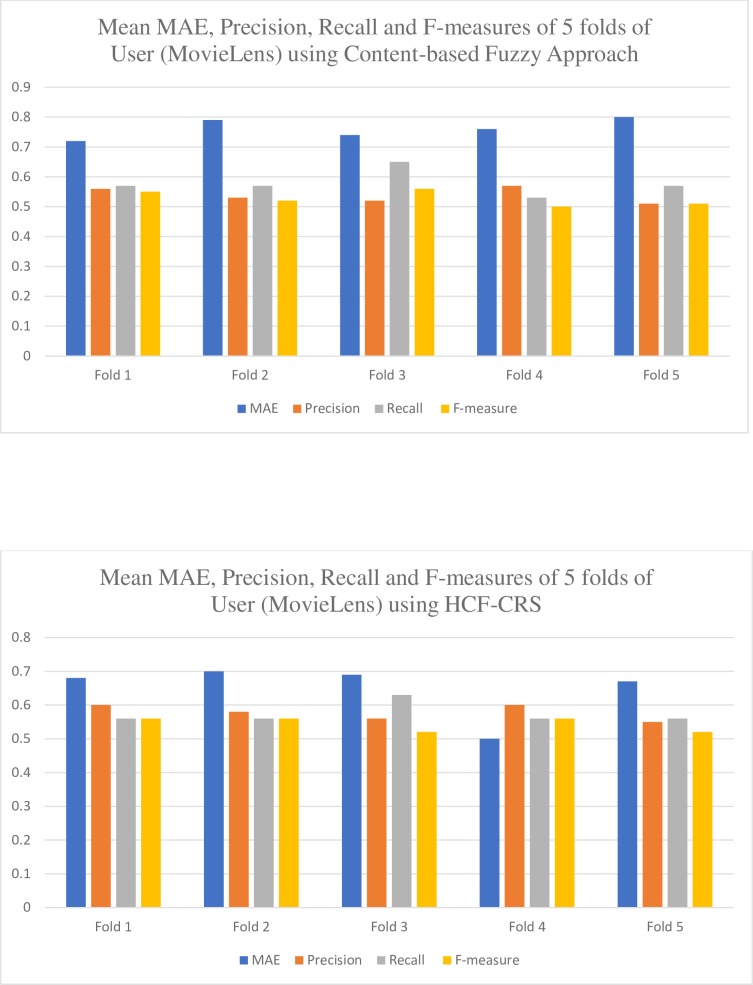
Evaluation results of using content-based Fuzzy approach and HCF-CRS on MovieLens.

The graphical illustration in Fig 7(A) and 7(B), shows the mean of MAE, precision, recall and f-measure of recommendations generated to five different fold of users from MovieLens dataset. Fig 7(A), demonstrates the results of using content-based fuzzy approach and it is observed that the result of almost all folds of user is almost the same but the fold 1 of users provides us with an MAE of 0.72 which is the best MAE with 0.56 precision, 0.57 recall and 0.55 f-measure. We can use the best mean we have obtained for the comparison of our content-based fuzzy approach.

Consider the Fig 7(B), it shows the results obtained from our HCF-CRS framework. It can be clearly observed that the mean MAE, precision, recall and f-measure are providing better results with the introduction of conformal prediction algorithm. The fold 4 of users gives the overall best results with an MAE of 0.5 which is the best MAE with 0.6 precision, 0.56 recall and 0.56 f-measure. We can use the best mean we have obtained for the comparison of our HCF-CRS framework.

**Fig 8:**
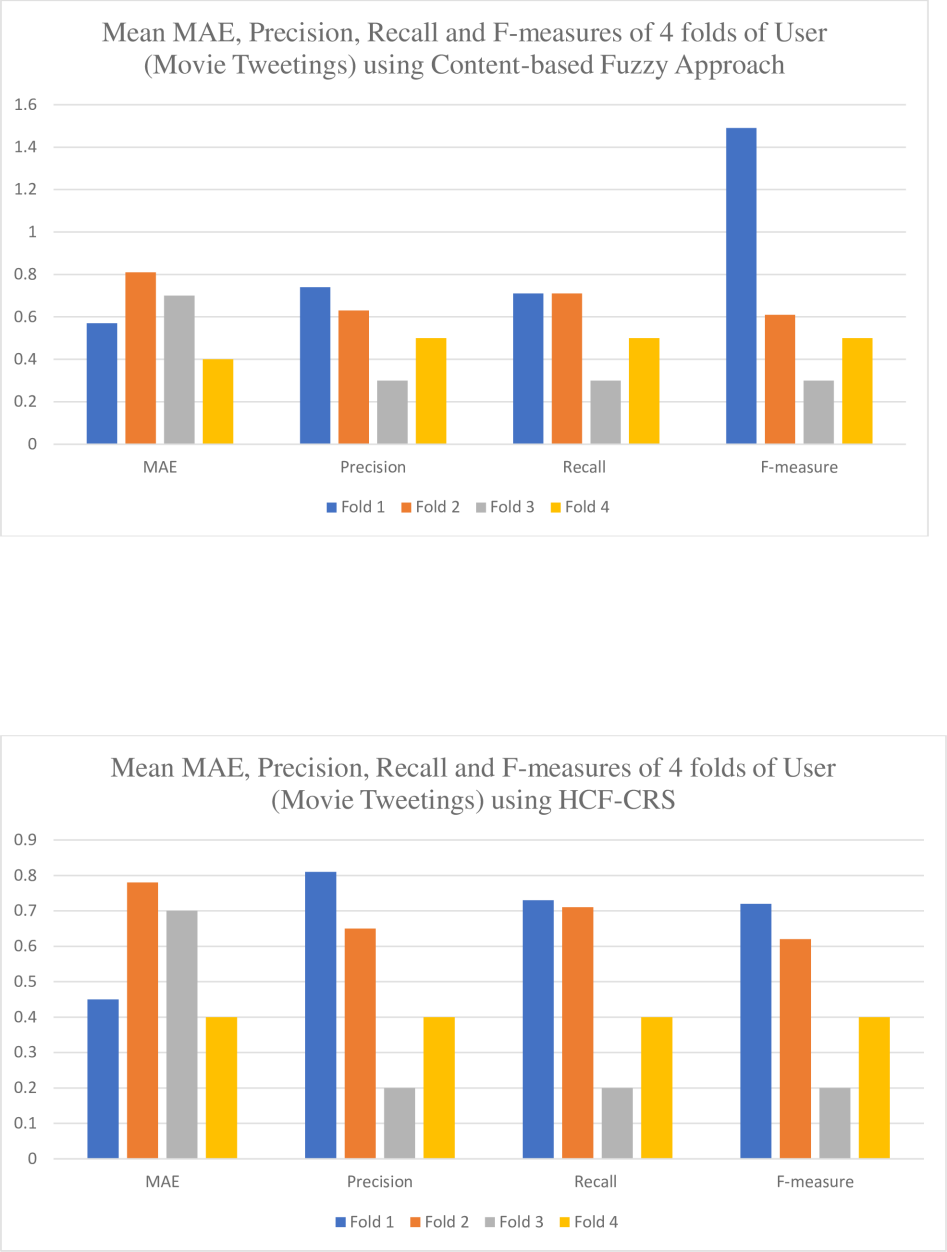
Evaluation results of using content-based Fuzzy approach and HCF-CRS on movie Tweetings.

Using the Movie Tweetings dataset, the users are randomly selected and divided into four folds of data with the two folds i.e. fold 3 and fold 4, including those users who have rated 2 or less than 2 items and the other 2 folds i.e. fold 1 and fold 2 having users who have rated more than 2 items and evaluated their results. The results are graphically illustrated in Fig 8(A) using the content-based fuzzy approach, it is observed that the fold 1 of users is giving us good results with 0.57 MAE, 0.74 precision, 0.71 recall and 1.4 f-measure while the fold 4 of users where the data is sparse we have achieved an MAE of 0.4 with precision 0.5, recall 0.5 and f-measure 0.5. Let us now consider the results for same folds of users using our proposed HCF-CRS framework illustrated in Fig 8(B), results of fold 1 of users is improved with an MAE of 0.45, a precision of 0.81 with recall and f-measure to be 0.73 and 0.72. Now considering the sparse folds of user, the fold 4 shows that the MAE is same as with using only a content based fuzzy algorithm i.e.0.4 but the values of precision, recall and f-measures are reduced. It can also be observed in fold 3 of users, the results are same with the same MAE and reduced precision using HCF-CRS. Therefore, we can say that with the sparse ratings, the content-based fuzzy approach works best as compared to the HCF-CRS framework.

### Comparison between proposed techniques

Table 3 displays the average and the best MAE, precision, recall and f-measure values we have obtained with our proposed techniques using the two datasets: MovieLens and Movie Tweetings. HCF-CRS framework is a hybrid approach and it can be observed from the table that it gives us better results as compared to when we are using an isolated technique e.g. content-based filtering or content-based fuzzy. An addition of conformal prediction algorithm has considerably improved the results. But, when the sparse matrix of Movie Tweetings dataset is perceived it is observed that the content-based fuzzy system works better with the movies having 1 or 2 ratings from the users. So, for the HCF-CRS framework to work best we need to have at least 3 ratings for each item. We would consider these results for the comparison with other state of the art recommender systems to prove the effectiveness and accuracy of our system.

**Table 3:** Average MAE of all three proposed techniques.

Datasets	Proposed Techniques	Average (Best) MAE	Average (Best) Precision	Average (Best) Recall	Average (Best) F-measure
**MOVIELENS 1M**	CBF	0.76	-	-	-
	Content based Fuzzy	0.72	0.56	0.57	0.55
	HCF-CRS	0.5	0.6	0.56	0.56
**MOVIE TWEETINGS**	Content based Fuzzy	0.57	0.74	0.71	1.49
	HCF-CRS	0.45	0.81	0.73	0.72
**(SPARSE)** **MOVIE TWEETINGS**	Fuzzy	0.4	0.5	0.5	0.5
	HCF-CRS	0.4	0.4	0.4	0.4

### Comparison with state of the art RS’s

To validate the effectiveness (reliability) and quality (accuracy) of our proposed HCF-CRS with other RS’s in literature. We should make sure that the experimental settings i.e. the evaluation metrics used, the format of dataset along with its ratings system is also the same. All the recommendation systems used for comparison have also used MovieLens or the Movie Tweetings dataset with the evaluation metrics MAE, Precision, Recall and F-measure. We have used the following recommendation systems in comparison with our proposed HCF-CRS framework.

1. A demographic recommender system [30], aims to solve the sparsity problem. This RS uses five different profiling approaches taking different user profile attributes in each approach to get the best of all. The experiments are conducted using three demographic attributes from MovieLens dataset. For comparison, the best improved value out of the specified best variants in each of five approaches with best MAE is considered.
2. The proposed recommender system in [31] totally depends on the demographic data of the user and if this information is not provided by the user then the algorithm will fail to work and additionally, the use of Pearson correlation coefficient cannot accurately measure the correlation.
3. A hybrid recommendation algorithm for bridging the movie feature and user interest for recommending movies to the user using the MovieLens dataset [32].
4. A hybrid RS combining the user-based and item-based Collaborative Filtering along with the Content based filtering. This hybrid recommendation method is proposed in a thesis report [33], which is considered to give efficient and accurate recommendations.
5. Using k-means clustering algorithm by implementing a cuckoo search optimization algorithm on a MovieLens dataset [39].
6. A hybrid model-based movie recommender system which utilizes the improved K-means clustering coupled with genetic algorithms (GAs) to partition transformed user space and a principle component analysis (PCA) to dense the movie space [40]. The best precision with neighborhood size 20 is 0.4 from 0.29 and the best recall value is 0.61 as the recommendation size grows with 20 recommendations. The mean MAE recorded is 0.78.
7. A Collaborative RS [55], which uses a multi-level recommendation method aims at providing high accuracy recommendations to users. An enough number of ratings is always required by the users to proceed with the proposed method.
8. We will compare of our proposed system with the results generated by the multi-level collaborative filtering recommendation method using MovieLens and Movie Tweetings dataset with the best evaluation results obtained.
9. A novel CBF method is proposed that uses a multi-attribute network that considers different attributes when calculating correlations to recommend items to users [56]. Similarities are measured between directly and indirectly linked items. The average precision and recall from the CBF multi-attribute technique is considered for comparison purposes.

The dataset used in the above defined recommender systems from literature for evaluation is the MovieLens [49] or the Movie Tweetings [50] dataset.

**Fig 9:**
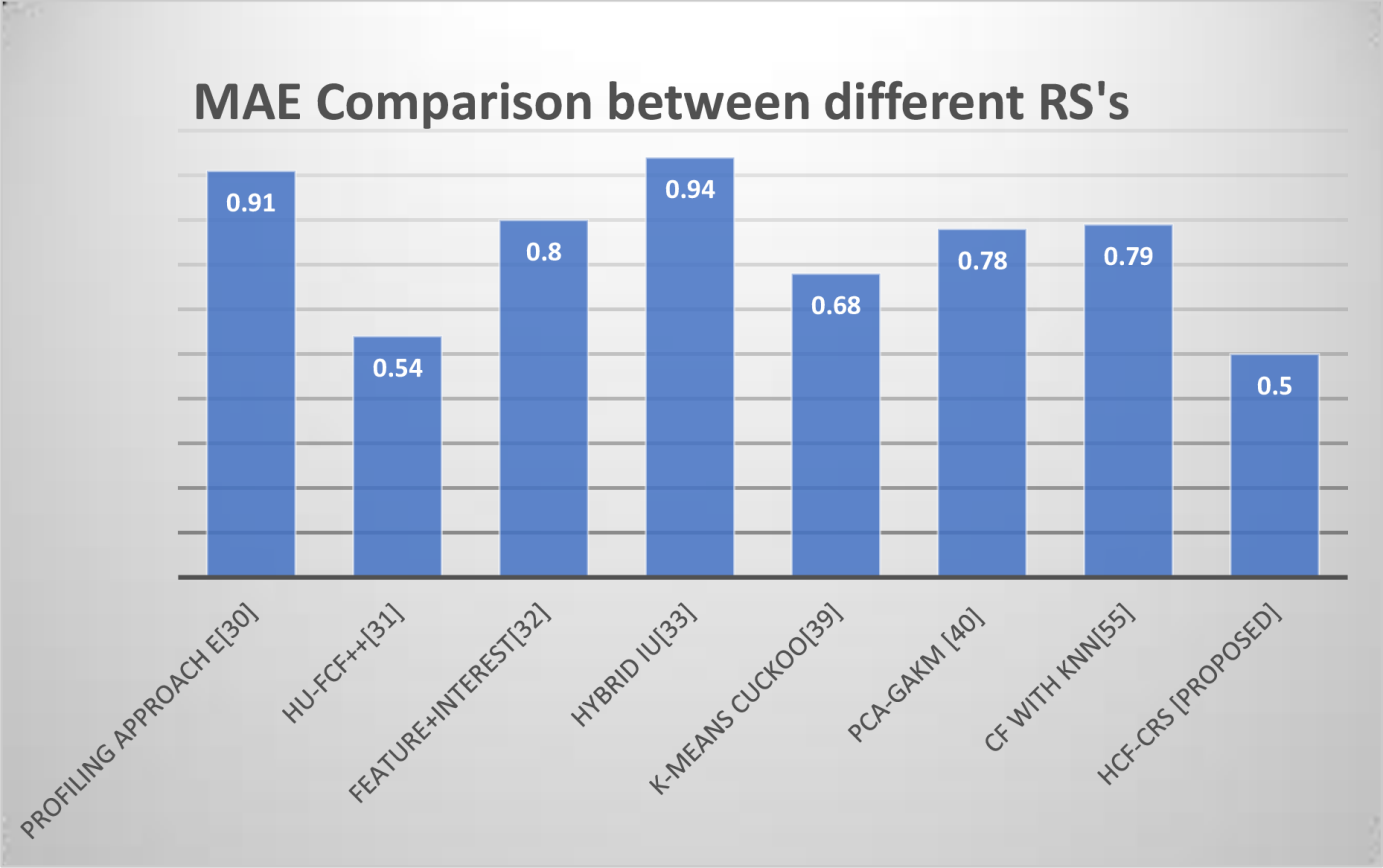
Comparison of Mean Absolute Error (MAE) between different RS’s using MovieLens dataset.

Fig 9. demonstrates the comparison graph of different MAE values of recommender systems from literature with our proposed HCF-CRS framework using MovieLens dataset.

Al Shamri in [30] used five profiling approaches for a demographic recommender system and it is observed that the best result is given by single attribute profiling approach E with a 0.91 MAE. Nikolaos in [55] used a CF method with different number of nearest neighbors and it is demonstrated that the MAE is higher when the number of neighbors are less e.g. with 10 nearest neighbors, the MAE is recorded as 0.81 and it gradually decreases to 0.79 when 40 nearest neighbors are considered, the figure remains the same up to 100 nearest neighbors. In [33], Seval Capraz proposed a content boosted CF approach using user based CF, item based CF and a hybrid of both. The results show that the hybrid method shows better results. Le Hoang son in [31], used 5 algorithms and used 8 folds of data to compute the accuracy. The results with the best accuracy from one of the folds are considered for comparison with our proposed RS. In [39], collaborative filtering with k-means cuckoo search optimization algorithm is applied and an MAE of 0.68 is achieved. PCA-GKM [40] has a calculated MAE of 0.78. It is observed that the proposed HCF-CRS framework outperforms all other RSs with improved accuracy.

**Fig 10:**
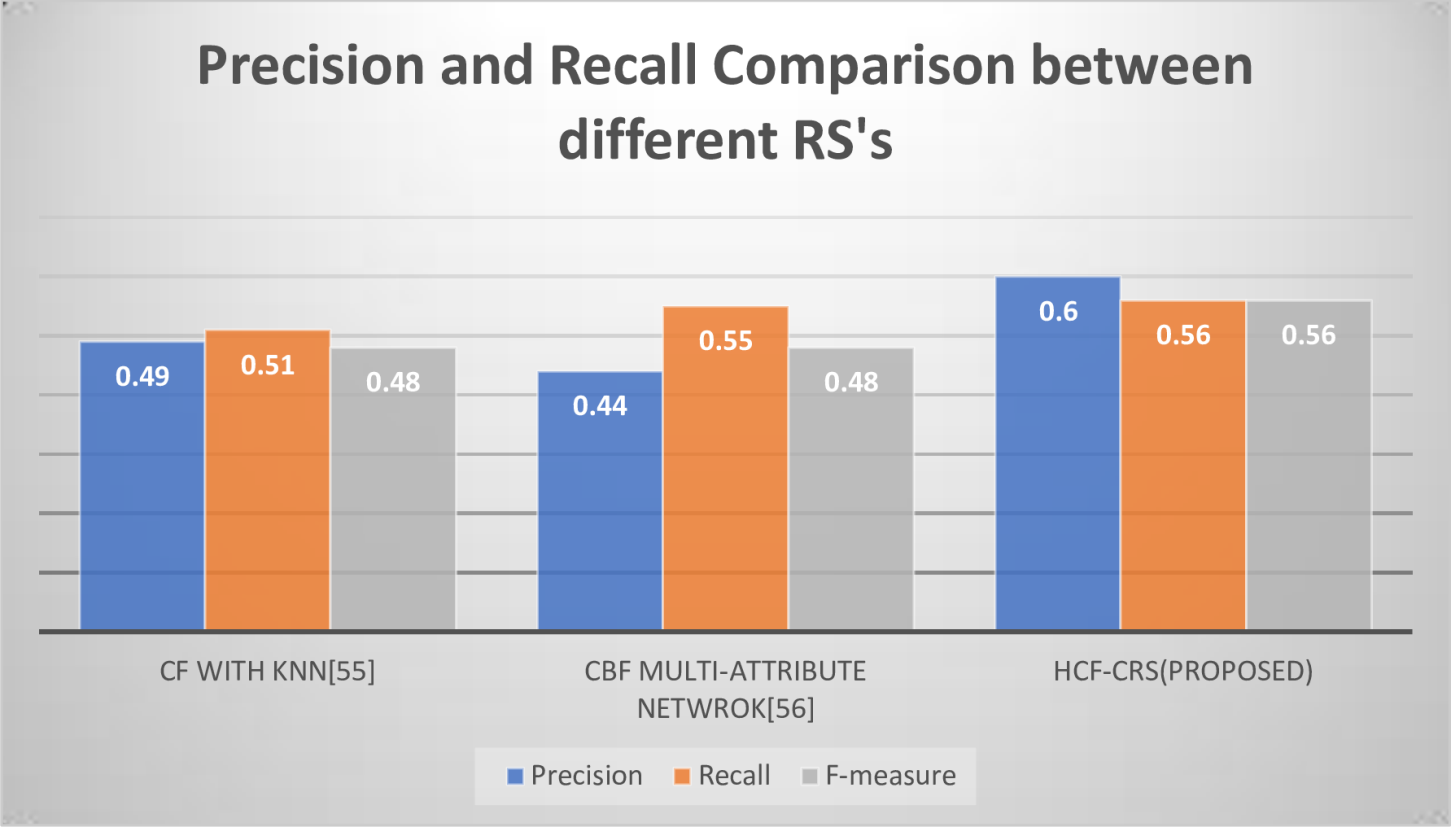
Comparison of precision, recall and F-measure between different RS’s using MovieLens dataset.

In Fig 10, the comparison graph of precision, recall and f-measure of three recommender systems from the literature are used for comparison. The f-measure, in most of the research has not been computed so we have focused on precision and recall. The best precision, recall and f-score values obtained with CF with different number of nearest neighbors [55] is 0.49, 0.51 and 0.48 respectively. Using PCA-GAKM [40], the precision is recorded as 0.4, the recall as 0.61and f-score is 0.46. For the multi-attribute network based RS, the precision is 0.44, recall is 0.55 and f-score is 0.48. These precision, recall and f-score values of RS’s from literature are compared with the precision of 0.6 and recall 0.56 with an f-measure of 0.56 to prove that our proposed HCF-CRS framework gives best results.

All the recommender systems used for comparison from literature have used MovieLens dataset for the evaluation purposes.

**Fig 11:**
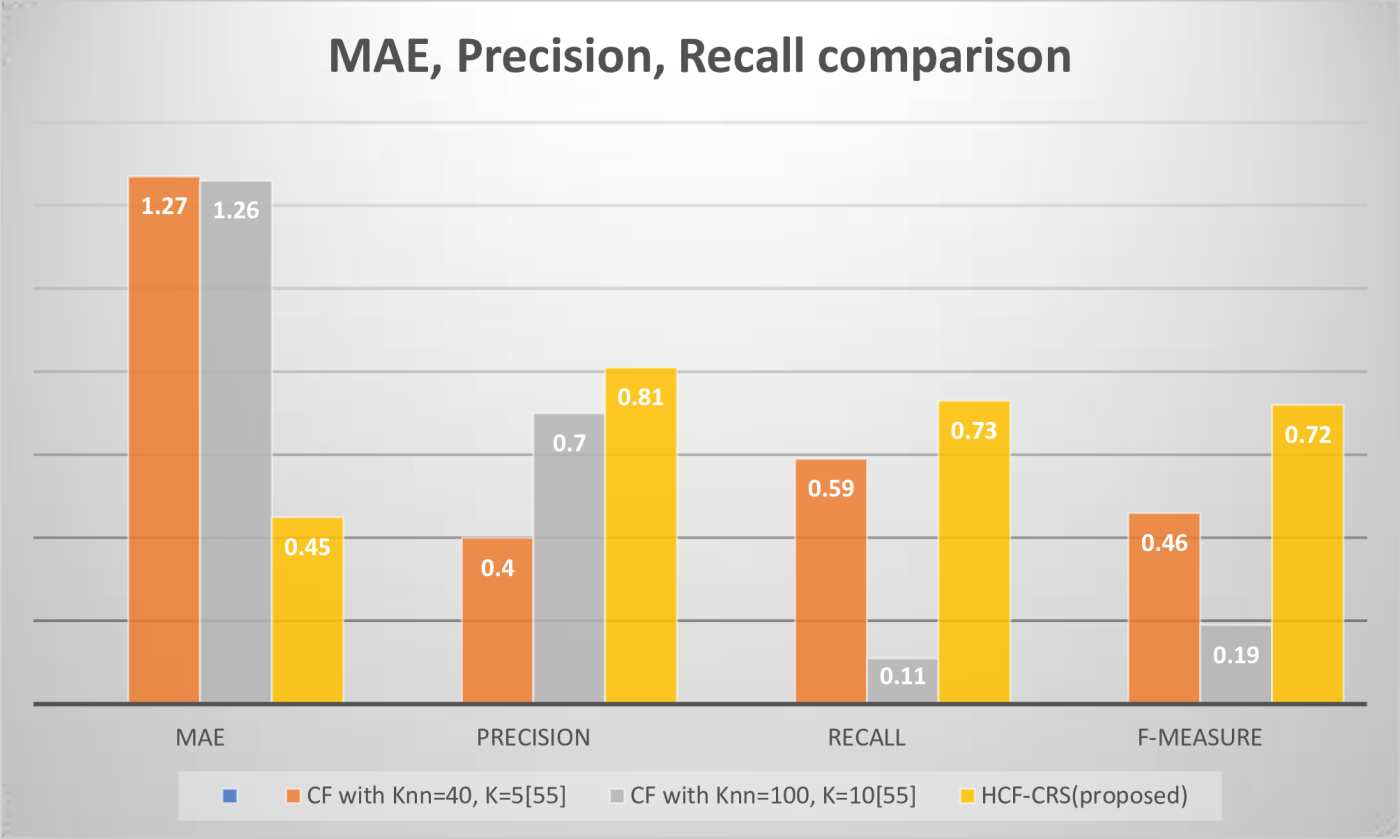
Comparison of Mean Absolute Error (MAE), precision, recall and F-measure between different RS’s using movie Tweetings dataset.

Fig 11. Demonstrates the evaluation performed on Movie Tweetings dataset with the graphical illustration of MAE, precision, recall and f-measure of one of the recommender systems from literature. It can be observed that our proposed HCF-CRS framework gives the best results. Hence, producing better quality recommendations.

## Discussion

Our proposed algorithms are tested on two datasets, MovieLens and Movie Tweetings which contains sparse ratings and it is experimentally proved that the conformal prediction framework works best with sufficient number of ratings while the content based fuzzy algorithm gives better results with the sparse dataset.

## Conclusion and future work

Recommender systems are widely adopted in several domains but is still contending with a challenge of data sparsity and unavailability of demographic data. The proposed HCF-CRS framework solves the data sparsity and lack of demographic information issue using the Content Based Filtering (CBF) technique, which creates a user profile by learning from the user history. A novel Fuzzy based technique is employed in our framework, which uses two features ‘similarity’ and ‘dissimilarity’ to compute the similarities and dissimilarities between the user profile and the movies data from the dataset to generate a list of users interested movies based on fuzzy rule set. HCF-CRS also initiates a conformal prediction technique to recommender systems by computing a non-conformity measure between the predicted ratings by proposed fuzzy system and the actual ratings from the movie dataset. The proposed Hybrid Content based Fuzzy Conformal Recommender System (HCF-CRS) generates a recommendation set with confidence level for each object to be recommended and the recommendation set is reduced with a confidence level higher than the set threshold. The reliability of the system is enhanced with the confidence being associated with each recommended item and thus the performance of the system is further improved. We have measured the performance of our approach using MAE, Precision, Recall and F-measure and compared with other state of the art recommender systems to validate its accuracy and reliability. For future work, the generation of highly personalized movie recommendations should be focused by taking into consideration other features such as context, trust or friendship. The accuracy or performance of the system can be improved by developing better non-conformity measurement method which adapt to recommender system. We can also consider, the speed (efficiency) of the system along with the accuracy.

## Supporting information

S1 File MovieLens dataset containing ratings. (ZIP)

S2 File MovieLens dataset containing movies. (CSV)

S3 File Movie Tweetings dataset containing ratings. (CSV)

S4 File Movie Tweetings dataset containing movies. (CSV)

S5 File Java code for recommendation of MovieLens movies. (DOCX)

S6 File Java code for recommendation of Movie Tweetings movies. (DOCX)

## References

[1] Katarya R, Verma OP. Recent developments in affective recommender systems. Physica A: Statistical Mechanics and its Applications. 2016;461:182–90.

[2] Li Y, Lu L, Xuefeng L. A hybrid collaborative filtering method for multiple-interests and multiple-content recommendation in E-Commerce. Expert Systems with Applications. 2005;28(1):67–77.

[3] Yoon VY, Hostler RE, Guo Z, Guimaraes T. Assessing the moderating effect of consumer product knowledge and online shopping experience on using recommendation agents for customer loyalty. Decision Support Systems. 2013;55(4):883–93.

[4] Vaz PC, Matos DMD, Martins B, Calado P. Improving a hybrid literary book recommendation system through author ranking. Proceedings of the 12th ACM/IEEE-CS joint conference on Digital Libraries—JCDL 12. 2012;

[5] Salakhutdinov R, Mnih A, Hinton G. Restricted Boltzmann machines for collaborative filtering. Proceedings of the 24th international conference on Machine learning—ICML 07. 2007;

[6] Oord A, Dieleman S, Schrauwen B. Deep Content-based Music Recommendation. Proceedings of the 26th International Conference on neural Information Processing. 2013;

[7] Hu X, Zhu W, Li Q. HCRS: A hybrid clothes recommender system based on user ratings and product features. Proceedings of the 2013 International Conference on management of e-commerce and e-government ICMEGG’13. 2013;

[8] Hirallal M. Recommender systems for e-shops—VU [Internet]. [cited 2018May23]. Available from: https://www.bing.com/cr?IG=97064862B80E415D9F4AEE0060C5E6FB&CID=33571D3E79176BD5053A16C678EA6A8F&rd=1&h=Tq3FBzytsTeDdU8sJURArUvwlNglXKC14BFX-g6eXmo&v=1&r=https://beta.vu.nl/nl/Images/werkstuk-hiralall_tcm235-202691.pdf&p=DevEx.LB.1,5374.1.2011;

[9] Schafer JB, Konstan J, Riedi J. Recommender systems in e-commerce. Proceedings of the 1st ACM conference on Electronic commerce—EC 99. 1999;

[10] Ekstrand MD. Collaborative Filtering Recommender Systems. Foundations and Trends® in Human–Computer Interaction. 2011;4(2):81–173.

[11] Su X, Khoshgoftaar TM. A Survey of Collaborative Filtering Techniques. Advances in Artificial Intelligence. 2009;2009:1–19.

[12] Wen Z. Recommendation System Based on Collaborative Filtering [Internet]. [cited 2018May23]. Available from: http://www.bing.com/cr?IG=44830C4D3018469EBA78A1C35B2C3FC8&CID=0D341C9F4EF663BF168117674F0B628B&rd=1&h=LfHMC1dMz3xvf_Lf7aptN8sySU8YmTQKrgJF5RFMX6k&v=1&r=http://cs229.stanford.edu/proj2008/Wen-RecommendationSystemBasedOnCollaborativeFiltering.pdf&p=DevEx.LB.1,5494.1.2008;

[13] Melville P, Sindhwani V. Recommender Systems. Encyclopedia of Machine Learning and Data Mining. 2017;:1056–66.

[14] Pazzani MJ, Billsus D. Content-Based Recommendation Systems. The Adaptive Web Lecture Notes in Computer Science. 2007;:325–41.

[15] Lops P, Gemmis MD, Semeraro G. Content-based Recommender Systems: State of the Art and Trends. Recommender Systems Handbook. 2010 5;:73–105.

[16] Degemmis M, Lops P, Semeraro G. A content-collaborative recommender that exploits WordNet-based user profiles for neighborhood formation. User Modeling and User-Adapted Interaction. 2007;17(3):217–55.

[17] Anand D, Bharadwaj KK. Enhancing Accuracy of Recommender System through Adaptive Similarity Measures Based on Hybrid Features. Intelligent Information and Database Systems Lecture Notes in Computer Science. 2010;:1–10.

[18] Liu H, Hu Z, Mian A, Tian H, Zhu X. A new user similarity model to improve the accuracy of collaborative filtering. Knowledge-Based Systems. 2014;56:156–66.

[19] Jannach D. Recommender systems: an introduction. New York: Cambridge University Press; 2012.

[20] Leben M. Applying Item-based and User-based Collaborative Filtering on the Netflix Data, Information Retrieval. 2008;

[21] Eckhardt A. Similarity of users’ (content-based) preference models for Collaborative filtering in few ratings scenario. Expert Systems with Applications. 2012;39(14):11511–6.

[22] Dias R. Combining Collaborative and Content-based Filtering to … [Internet]. 2004 [cited 2018May23]. Available from: http://www.bing.com/cr?IG=25900AED8D934D6092A5E1DB33216F8C&CID=28988CFDDB39638408CC8705DAE7621F&rd=1&h=c_Ny2Y0XEZ2CLOuyjoskBMtYZUbbF2EiIEUNScRXA_8&v=1&r=http://www.inf.ufrgs.br/bdi/wp-content/uploads/MastersDissertationRobertoTorres.pdf&p=DevEx.LB.1,5513.1.2004;

[23] Campos LMD, Fernández-Luna JM, Huete JF, Rueda-Morales MA. Combining content-based and collaborative recommendations: A hybrid approach based on Bayesian networks. International Journal of Approximate Reasoning. 2010;51(7):785–99.

[24] Lika B, Kolomvatsos K, Hadjiefthymiades S. Facing the cold start problem in recommender systems. Expert Systems with Applications. 2014;41(4):2065–73.

[25] Zhang Z-K, Liu C, Zhang Y-C, Zhou T. Solving the cold-start problem in recommender systems with social tags. EPL (Europhysics Letters). 2010 1;92(2):28002.

[26] Shafer G., Vovk V. Machine Learning Research. Rutgers University and University of London 2008; 9.

[27] Linusson H, Johansson U. Conformal Prediction—A Tiny Tutorial on Predicting with … [Internet]. [cited 2018May23]. Available from: https://www.bing.com/cr?IG=62FAFEB4770C460FBB07B22F1FA34B12&CID=3804B6F9F20D69001668BD01F3F068EF&rd=1&h=WJE9qD5Nde2BhP6S7a9KAeIB6U4P0gKz-lTKWjxycnY&v=1&r=https://people.dsv.su.se/~henke/DSWS/johansson.pdf&p=DevEx.LB.1,5497.1.2014;

[28] Nouretdinov I, Gammerman A, Qi Y, Klein-Seetharaman J. Determining Confidence Of Predicted Interactions Between Hiv-1 And Human Proteins Using Conformal Method. Biocomputing 2012 2011; PMC3249613 22174286

[29] Papadopoulos H. Inductive Conformal Prediction: Theory and Application to Neural Networks. Tools in Artificial Intelligence. 2008; 10.1016/j.engappai.2007.04.007

[30] Al-Shamri MYH. User profiling approaches for demographic recommender systems. Knowledge-Based Systems. 2016;100:175–87.

[31] Son LH. HU-FCF: A novel hybrid method for the new user cold-start problem in recommender systems. Engineering Applications of Artificial Intelligence. 2015;41:207–22.

[32] Li J, Xu W, Wan W, Sun J. Movie recommendation based on bridging movie feature and user interest. Journal of Computational Science. 2018;26:128–34.

[33] Çapraz S. A Content Boosted Hybrid Recommender System [Internet]. Academia.edu. [cited 2018May24]. Available from: http://www.academia.edu/34040823/A_Content_Boosted_Hybrid_Recommender_System.2016;

[34] Akcayol MA, Utku A, Aydoğan E, Mutlu B. A weighted multi-attribute-based recommender system using extended user behavior analysis. Electronic Commerce Research and Applications. 2018;28:86–93

[35] Shani G, Gunawardana A. Evaluating Recommendation Systems. Recommender Systems Handbook. 2010 5;:257–97.

[36] Mazurowski MA. Estimating confidence of individual rating predictions in collaborative filtering recommender systems. Expert Systems with Applications. 2013;40(10):3847–57.

[37] Koren Y, Sill J. OrdRec. Proceedings of the fifth ACM conference on Recommender systems—RecSys 11. 2011;

[38] Tejeda-Lorente Á, Porcel C, Peis E, Sanz R, Herrera-Viedma E. A quality based recommender system to disseminate information in a university digital library. Information Sciences. 2014;261:52–69.

[39] Katarya R, Verma OP. An effective collaborative movie recommender system with cuckoo search. Egyptian Informatics Journal. 2017;18(2):105–12.

[40] Wang Z, Yu X, Feng N, Wang Z. An improved collaborative movie recommendation system using computational intelligence. Journal of Visual Languages & Computing. 2014;25(6):667–75.

[41] Himabindu TV, Padmanabhan V, Pujari AK. Conformal matrix factorization based recommender system. Information Sciences. 2018; 10.1016/j.ins.2017.08.093

[42] Pablo CI, Alcala-Fdez J. jFuzzyLogic: a Java Library to Design Fuzzy Logic … [Internet]. [cited 2018May23]. Available from: http://www.bing.com/cr?IG=1025E9BD6CB54445886E166D6F75ACF9&CID=01D6F2C0294A6A5F299AF93828B76B64&rd=1&h=LP4fOHER-c0FkyuOqQHqS1txMysKenbb9eWa3ieO7vM&v=1&r=http://jfuzzylogic.sourceforge.net/html/pdf/Cingolani_Alcala-Fdez_jFuzzyLogic_2013_IJCIS.pdf&p=DevEx.LB.1,5496.1

[43] Cingolani P, Alcala-Fdez J. jFuzzyLogic: a robust and flexible Fuzzy-Logic inference system language implementation. 2012 IEEE International Conference on Fuzzy Systems. 2012;

[44] Beale M, Demuth H. Fuzzy Logic Toolbox [Internet]. MATLAB & Simulink. [cited 2018May23]. Available from: https://www.mathworks.com/products/fuzzy-logic.html

[45] Lee J, Sun M, Lebanon G. Comparative Study of Collaborative Filtering Algorithms. Proceedings of the International Conference on Knowledge Discovery and Information Retrieval. 2012;

[46] Ekstrand MD. Collaborative Filtering Recommender Systems. Foundations and Trends® in Human–Computer Interaction. 2011;4(2):81–173.

[47] Kumar V, Pujari AK, Sahu SK, Kagita VR, Padmanabhan V. Collaborative filtering using multiple binary maximum margin matrix factorizations. Information Sciences. 2017;380:1–11.

[48] V. SD, Kagita VR, Pujari AK, Padmanabhan V. Collaborative filtering by PSO-based MMMF. 2014 IEEE International Conference on Systems, Man, and Cybernetics (SMC). 2014;

[49] Kagita VR, Pujari AK, Padmanabhan V, Sahu SK, Kumar V. Conformal recommender system. Information Sciences. 2017;405:157–74.

[50] Harper, FM. The MovieLens Datasets: History and Context [Internet]. Communications of the ACM. ACM; [cited 2018May23]. Available from: https://dl.acm.org/citation.cfm?doid=2866565.2827872. 2015;

[51] Dooms S, Martens L. "Harvesting movie ratings from structured data in social media" by Simon Dooms and Luc Martens with Ching-man Au Yeung as coordinator. ACM SIGWEB Newsletter. 2014 1;(Winter):1–5.

[52] Chai T, Draxler RR. Root mean square error (RMSE) or mean absolute error (MAE … [Internet]. [cited 2018May23]. Available from: http://www.bing.com/cr?IG=DC1A90B9CFAB4EC697B4CFDA5F1008B8&CID=28B4AB78E6E86E5A35C7A080E7156F7D&rd=1&h=324FoQja37240Ol2ATJxwRF-JR3Dz0pGLlb2gxHquIU&v=1&r=http://www.geosci-model-dev.net/7/1247/2014/gmd-7-1247-2014.pdf&p=DevEx.LB.1,5485.1.2014;

[53] Chapter 07—Evaluating recommender systems [Internet]. [cited 2018May23]. Available from: https://www.bing.com/cr?IG=DAA3ED6CCF47410B8998528C5AFCA222&CID=20FDFCB6EBD560C92505F74EEA28617E&rd=1&h=EfXMQvpaRzK02YYL6BiYvACeIO1n3SjrO3OHizUUBa0&v=1&r=https://arxiv.org/pdf/1503.06410&p=DevEx.LB.1,5487.1

[54] Powers DMW. What theF-measure doesn’t measure—arXiv [Internet]. [cited 2018May23]. Available from: https://www.bing.com/cr?IG=DAA3ED6CCF47410B8998528C5AFCA222&CID=20FDFCB6EBD560C92505F74EEA28617E&rd=1&h=EfXMQvpaRzK02YYL6BiYvACeIO1n3SjrO3OHizUUBa0&v=1&r=https://arxiv.org/pdf/1503.06410&p=DevEx.LB.1,5487.1

[55] Polatidis N, Georgiadis CK. A multi-level collaborative filtering method that improves recommendations. Expert Systems with Applications. 2016;48:100–10.

[56] Son J, Kim SB. Content-based filtering for recommendation systems using multi-attribute networks. Expert Systems with Applications. 2017;89:404–12.

