# Supplementary material for: HCF-CRS: A Hybrid Content based Fuzzy Conformal Recommender System for providing recommendations with confidence
Source: PLoS One. 2018 Oct 9;13(10):e0204849. doi: 10.1371/journal.pone.0204849 (PMC6177139; doi:10.1371/journal.pone.0204849)
Supplement: S6 File — (DOCX) [file pone.0204849.s006.docx]

/**

*

* @author sundussultan

*/

/*

* To change this license header, choose License Headers in Project Properties.

* To change this template file, choose Tools | Templates

* and open the template in the editor.

*/

import java.io.BufferedReader;

import java.io.BufferedWriter;

import java.io.FileReader;

import java.io.FileWriter;

import java.io.IOException;

import java.io.InputStreamReader;

import java.util.ArrayList;

import java.util.Arrays;

import java.util.Collections;

import static java.util.Collections.list;

import java.util.Comparator;

import java.util.HashMap;

import java.util.HashSet;

import java.util.Iterator;

import java.util.List;

import java.util.Map;

import java.util.NavigableMap;

import java.util.NavigableSet;

import java.util.Set;

import java.util.StringTokenizer;

import java.util.TreeMap;

import java.util.Map.Entry;

import java.util.concurrent.ConcurrentHashMap;

import java.math.*;

import net.sourceforge.jFuzzyLogic.FIS;

import org.apache.commons.math3.util.Precision;

/**

*

* @author HP

*/

public class MovieTweetings {

/**

* @param args the command line arguments

*/

public static void main(String[] args) throws IOException {

try

{

//create BufferedReader to read csv file

BufferedReader stream = new BufferedReader( new FileReader("result2.csv"));

BufferedReader stream2 = null;

BufferedReader stream3 = null; // using this reader for Evaluation Purposes

BufferedReader Conformalstream = null;

List<String> TagTokens = new ArrayList<String>();// tokens to find the frequency of each movie genre

List<String> TagTokens2 = new ArrayList<String>(); // tokens of movie genre in db (user interested)

List<List<String>> TagTokens3 = new ArrayList<List<String>>(); // tokens mostly interested to user from movie genre

List<String> TagTokens4 = new ArrayList<String>(); // contains movie genre user already watched

List<String> movieGenre = new ArrayList<String>(); // contains user profile movie genre

List<List<String>> movieGenre2 = new ArrayList<List<String>>(); // contains all movie genre in db

Set<List<String>> hs = new HashSet<>(); // no need of order in case of hashmap

String line = "";

String line2 = "";

String line3="";

String line4="";

String line5="";

String confline="";

String splitBy=",";

String splitBy2=",";

StringTokenizer st = null;

StringTokenizer st2 = null;

StringTokenizer st4 = null;

Map<String, Integer> wordCount = new HashMap<String, Integer>();

Map<String, Double> FinalScore = new HashMap<String, Double>();

ArrayList<HashMap<String, Double>> removeDupli=new ArrayList<>();

TreeMap<Integer, String> treemap= new TreeMap<Integer, String>(Collections.reverseOrder());

Map<String, Integer> treemap2= new HashMap<String, Integer>();

double mae1=0.00;

double confmae1=0.00;

int TP2=0; //true positive

int FP2=0; //false positive

int FN2=0; //false negative

int TN2=0; //true negative

double F1measure2 = 0.00;

int TP=0; //true positive

int FP=0; //false positive

int FN=0; //false negative

int TN=0; //true negative

double F1measure = 0.00;

int maeCounter=0;

int confmaeCounter=0;

int counter=0;

double predictedRating4;

InputStreamReader inputStreamReader = new InputStreamReader(System.in);

BufferedReader reader = new BufferedReader(inputStreamReader);

System.out.println("Enter User ID:");

String userID = reader.readLine();

//System.out.println("Hello "+name);

// PVALUE VARIABLES DECLARATION

// int lineNumber = 0, tokenNumber = 0;

//read comma separated file line by line

while( (line = stream.readLine()) != null) { //reading result.csv file

String[] readCol= line.split(",");

// lineNumber++;

// System.out.println("read column 2...."+readCol[2]);

//break comma separated line using ","

st = new StringTokenizer(readCol[2], "|"); //column 2 contains movie genre

// System.out.println(readCol[0]);

// THE USER NO. TO SUGGEST HIM MOVIES

if (readCol[0].equals(userID)){ //column 0 contains user IDs

while(st.hasMoreTokens())

{

TagTokens.add(st.nextToken()); //tokens of movie genre

} // end of making tokens

// System.out.println("Tag Tokens......" +TagTokens); //movie genre of given user ID

//reset token number

} // if end

}// end of reading lines (while loop)

// System.out.println("Tokens : "+TagTokens);

for(String word: TagTokens) {

Integer count = wordCount.get(word);

wordCount.put(word, (count==null) ? 1 : count+1);

}

System.out.println("Frequency of Words " +wordCount);

for (Map.Entry<String, Integer> entry : wordCount.entrySet()) // Get the most frequent item (having largest value)

{

treemap.put(entry.getValue(),entry.getKey()); //treemap contains the most frequent value

}// end for loop

Set set = treemap.entrySet();

Iterator i = set.iterator();

// Display elements

/* while(i.hasNext()) {

Map.Entry me = (Map.Entry)i.next();

System.out.print(me.getKey() + ": ");

System.out.println(me.getValue());

}*/

Map.Entry me;

// GET THE TOP 3 MOST INTERESTED MOVIE GENRES FOR THE SPECIFIC USER

// This makes User Profile

System.out.println("THE USER PROFILE CONTAINS.....FOLLOWING GENRE");

for(int k=0;k<1;k++){

i.hasNext();

me=(Map.Entry)i.next();

System.out.print(me +"|");

movieGenre.add(me.getValue().toString()); //movieGenre is the User Profile containing the mostly watched movie Genre

} // end for loop

int count=1;

int index=0;

for (int in=0;in<1;in++)

{

stream2 = new BufferedReader( new FileReader("mymovies2.csv"));

// RETRIEVE MOVIES FROM MYMOVIES.CSV File having the Genres retrieved above

while ((line2 = stream2.readLine()) != null){ //stream2 is mymovies.csv file

String[] readCol2= line2.split(splitBy2);

//movieGenre is the USER PRoFILE. so get genre at index 0 of user profile and match with all movies in mymovies.csv file

if (readCol2[4].contains(movieGenre.get(index))) //if the movie genre in mymovie.csv file contains the following genre

{

// System.out.println(count+ ": Suggested movie :"+readCol2[1] +"Movie Code: "+readCol2[0]);

movieGenre2.clear();

movieGenre2.add(Arrays.asList(readCol2[0])); // contains movie codes, add movie codes from mymovies.csv file into movieGenre2

String movieCode=movieGenre2.toString();

count++;

st2 = new StringTokenizer(readCol2[4], "|");

while(st2.hasMoreTokens())

{ //TagTokens2.clear();

TagTokens2.add(st2.nextToken());

// System.out.println(TagTokens2);

} // end of making tokens

// System.out.println("Each Movie Genre"+TagTokens2);

int movieKeywords= TagTokens2.size();

// System.out.println(TagTokens2+".....Tag Tokens 2....." +movieKeywords);

// System.out.println("Movie Keywords "+movieKeywords);

// System.out.println("tag token size "+TagTokens2.size());

// System.out.println("User Profile movie genre"+movieGenre);

TagTokens2.retainAll(movieGenre);// calculates the intersection b/w user profile movie genre and movie genre from mymovies.csv file

// System.out.println(TagTokens2.size()+" : intersection "+TagTokens2);

// System.out.println("Intersection Size "+.size());

// (ONLY for CONTENT BASED) LETS PUT THE SIMILARITY FORMULA TO GET THE SCORE OF EACH MOVIE

int userprofileKeywords=movieGenre.size();

// System.out.println("user profile keywords "+userprofileKeywords);

int denominator=movieKeywords+userprofileKeywords;

// double movieScore= (TagTokens2.size()/denominator);

// movieScore=movieScore*2;

// +++ +++++++***************FUZZY LOGIC (similarity and dissimilarity) Features +++++++++++***********

int similarity= TagTokens2.size();

int dissimilarity= denominator-(similarity*2);

// System.out.println("similarity... :"+similarity+" dissimilarity: "+dissimilarity);

String fileName = "features.fcl";

FIS fis = FIS.load(fileName,true);

// Error while loading?

if( fis == null ) {

System.err.println("Can't load file: '"

+ fileName + "'");

//return;

}

// Show

// fis.chart();

// Set inputs

fis.setVariable("Similarity", similarity);

fis.setVariable("Dissimilarity",dissimilarity );

// System.out.println("Similarity score"+similarity+" Dissimilarity score"+dissimilarity);

// Evaluate

fis.evaluate(); // using the fuzzy rules

// to know the system's output

double value= fis.getVariable("expectedRating").getLatestDefuzzifiedValue();

// System.out.println("the latest defuzzified value is "+Math.round(value));

double predictedRating= Math.round(value);

//Final Score of each movie in a Hash Map with its Movie Code

// FinalScore.put(movieCode, movieScore);

// System.out.println("Final Map : " + FinalScore);

// THE PREDICTED RATING OF MOVIES USING FUZZY LOGIC IS STORED IN FINAL SCORE MAP

FinalScore.put(movieCode, predictedRating); //contains the movie code with the predicted fuzzy RATING

} // end of if

TagTokens2.clear();

} //end while reading mymovies.csv file

index++;

} //end for

// Remove DUPLICATE from MAP (Final Score)

System.out.println("\n After removing duplicate values ");

for(String key1:FinalScore.keySet()){

for(Object key2:FinalScore.keySet()){

if(!key1.toString().equals(key2.toString())){

Double x=FinalScore.get(key1);

Double y=FinalScore.get(key2);

if(x==y){

FinalScore.remove(key2);

} // end inner if

} // end if

} //end inner for

} //end outer for

System.out.println("Final Score Map contains....."+FinalScore+"\n");

// ---------------------xxxxxxxxxxx CONFORMAL PREDICTION xxxxxxxxxxxxx------------------

// CALCULATING THE NON CONFORMITY SCORES OF ALL THE MOVIES WATCHED BY THE GIVEN USER ID

// read the myRatings.csv file for non conformity scores of each watched movie by given user id

BufferedReader stream4 = new BufferedReader( new FileReader("myRatings2.csv")); // using this reader for reading result2.csv file

while( (line4 = stream4.readLine()) != null) //reading myRatings.csv file

{

String[] readCol4= line4.split(",");

// lineNumber++;

//break comma separated line using ","

// THE USER ID NO.

if (readCol4[0].equals(userID)){ //column 0 contains user IDs

counter++;

String y= readCol4[1].toString(); //moviecode

if (FinalScore.get("[["+y+"]]")!=null) // y contains the movie code from myRatings file

{

predictedRating4= FinalScore.get("[["+y+"]]"); //getting the predicted rating from finalscore map

}

else predictedRating4=0.0;

int actualRating4= Integer.parseInt(readCol4[2]); // reading the actual rating from myRatings.csv file

int pR = (int)predictedRating4;

int nonConformityScore= Math.abs(pR-actualRating4) ;

treemap2.put(y, nonConformityScore);

} // end if equalling given user id

} // end of reading file myRatings.csv

/* read the movie code of each line

and compare it with the movie code from finalscore map

subtract the actual rating from ratings file from the predicted rating in finalscore map

get the non conformity score

save non conformity score as e.g. 1 , 1, 0, 4,

count the total number of movies rated by that user

next p value

*/

// SORT THE LIST OF MOVIE CODES WITH SCORE in DESCENDING ORDER

Set<Entry<String, Double>> set2 = FinalScore.entrySet();

List<Entry<String, Double>> list = new ArrayList<Entry<String, Double>>(set2);

Collections.sort( list, new Comparator<Map.Entry<String, Double>>()

{

public int compare( Map.Entry<String, Double> o1, Map.Entry<String, Double> o2 )

{

return (o2.getValue()).compareTo( o1.getValue() );

}

} );

for(Map.Entry<String, Double> entry:list){

double rating=entry.getValue();

int predictedRating= (int)rating;

System.out.println(entry.getKey()+" ==Predicted Rating== "+predictedRating);

String recomMovie= entry.getKey();

// CALCULATE THE P VALUE OF EACH RECOMMENDED MOVIE HERE

// ++++++++++++++++++++++++++P_VALUE+++++++++++++++++++++++++++++++++++

BufferedReader stream5 = new BufferedReader( new FileReader("AverageMovieRatings2.csv"));

begin2: while( (line5 = stream5.readLine()) != null) //reading AverageMovieRatings.csv file

{

String[] readCol5= line5.split(",");

if (recomMovie.equals("[["+readCol5[0]+"]]")){

int avgRating = Integer.parseInt(readCol5[1]);

int pNumerator= Math.abs(predictedRating-avgRating);

int add=0;

// CALCULATING THE 1st P_VALUE

for(Integer value: treemap2.values()){ //values in tree map greater than or equal to 1/2/3 etc.

if (value>pNumerator || value.equals(pNumerator)){

add=add+1;

} //end if

}// end for

// System.out.println("add1....."+add1);

double p_value= (double)add/(counter+1);

//System.out.println("counter....."+counter);

// Precision.round(p_value1, 2);

System.out.println("PVALUE....."+Precision.round(p_value, 2)); // rounding the p value to 2 decimal points

//+++++++++++++++= calculate the MAE after getting the Pvalues +++++++++++++++++++++++++++

if (p_value>0.45) { // bound is set at 0.45 confidence level where (significance level) e=0.55

Conformalstream= new BufferedReader(new FileReader("myRatings2.csv"));

begin: while ((confline=Conformalstream.readLine())!=null){

String[] confreadCol = confline.split(splitBy2);

// System.out.println("HERE reading....................... "+readCol3[0]);

if (confreadCol[0].equals(userID)){

if(entry.getKey().equals("[["+confreadCol[1]+"]]")){

System.out.println("INSIDE NOW for CRS....................... "+confreadCol[1]+"and"+entry.getKey());

int actualRating1= Integer.parseInt(confreadCol[2]);

int subtraction1= actualRating1-predictedRating;

// CONFUSION MATRIX FOR CALCULATING PRECISION AND RECALL MEASURE

if (subtraction1 == 0) {TP=TP+1;}

else if (subtraction1 < 0) {FP=FP+1;}

else if (subtraction1 > 0) {FN=FN+1;}

else if (subtraction1 == 0 && actualRating1<3 || predictedRating <3) {FN=FN+1;}

System.out.println("Absolute Difference :"+Math.abs(subtraction1));

System.out.println("TP :"+TP+ "FP :"+FP +"FN :"+FN);

double absDiff1= Math.abs(subtraction1);

// Calculating the Non-Conformity Score of each watched movie by the specific user ID..............

confmae1= confmae1+absDiff1; // caculating the total error

confmaeCounter++;

break;

} //inner if clause

else continue begin ;

}

else

continue begin;

} // end while

}

break;

}

else continue begin2;

}// end of while reading file

/* read result.csv file where user id is (6000) check similarity percentage

and add the movies in a list. count them and calculate their non confirmity

and p value

*/

//+++++++++++++++EVALUATION++++++

stream3= new BufferedReader(new FileReader("myRatings2.csv"));

begin: while ((line3=stream3.readLine())!=null){

String[] readCol3 = line3.split(splitBy2);

// System.out.println("HERE reading....................... "+readCol3[0]);

if (readCol3[0].equals(userID)){

// System.out.println("INSIDE NOW....................... "+readCol3[1]+"and"+entry.getKey());

if(entry.getKey().equals("[["+readCol3[1]+"]]")){

System.out.println("INSIDE NOW....................... "+readCol3[1]+"and"+entry.getKey());

int actualRating= Integer.parseInt(readCol3[2]);

int subtraction= actualRating-predictedRating;

System.out.println("Absolute Difference :"+Math.abs(subtraction));

if (subtraction == 0) {TP2=TP2+1;}

else if (subtraction < 0) {FP2=FP2+1;}

else if (subtraction > 0) {FN2=FN2+1;}

else if (subtraction == 0 && actualRating<3 || predictedRating <3) {FN2=FN2+1;}

System.out.println("TP2 :"+TP2+ "FP :"+FP2 +"FN2 :"+FN2);

double absDiff= Math.abs(subtraction);

// Calculating the Non-Conformity Score of each watched movie by the specific user ID..............

mae1= mae1+absDiff; // caculating the total error

maeCounter++;

break;

} //inner if clause

else continue begin ;

}

else

continue begin;

} // end while

} //end for loop

// System.out.println("TREEMAP of Non Conformity Score...."+treemap2);

System.out.println("TCounter ===="+counter);

System.out.println("TreeMap2 ===="+treemap2); // contains the non-conformity score of user id movies

System.out.println("The mae1 ===="+mae1+ "the maeCounter is "+maeCounter);

double mae= mae1/maeCounter;

System.out.println("The MEAN ABSOLUTE ERROR ===="+mae);

System.out.println("The CRS MAE ===="+confmae1+ "the maeCounter is "+confmaeCounter);

double confmae= confmae1/confmaeCounter;

System.out.println("The MEAN ABSOLUTE ERROR With COnformal Prediction===="+confmae);

//MEASUREMENT OF PRECISION , RECALL AND F1 MEASURE VALUES

double precision2 = (double) TP2/ (double)(TP2+FP2);

System.out.println("Precision is "+precision2);

double recall2= (double)TP2/ (double) (TP2+FN2);

System.out.println("Recall is "+recall2);

F1measure2= 2* (precision2*recall2)/(precision2+recall2);

System.out.println("F1 measure is "+F1measure2);

//MEASUREMENT OF PRECISION , RECALL AND F1 MEASURE VALUES AFTER APPLYING CONFORMAL PREDICTION

double precision = (double) TP/ (double)(TP+FP);

System.out.println("Precision with Conformal is "+precision);

double recall= (double)TP/ (double) (TP+FN);

System.out.println("Recall with Conformal "+recall);

F1measure= 2* (precision*recall)/(precision+recall);

System.out.println("F1 measure with conformal is "+F1measure);

} //end of try clause

catch(Exception e)

{

e.printStackTrace();

System.out.println("Exception while reading csv file: " + e );

} //end of catch clause

} // main end

} // end of class
